# Investigating causality and shared genetic architecture between body mass index and cognitive function: a genome-wide cross-trait analysis and bi-directional Mendelian randomization study

**DOI:** 10.3389/fnagi.2024.1466799

**Published:** 2024-10-16

**Authors:** Mingyi Chen, Xiaoxin Xu, Fang Wang, Xiaohong Xu

**Affiliations:** ^1^Department of Neurology and Stroke Center, The First Affiliated Hospital of Jinan University, Guangzhou, Guangdong, China; ^2^Clinical Neuroscience Institute, The First Affiliated Hospital of Jinan University, Guangzhou, Guangdong, China

**Keywords:** cognitive function, body mass index, bi-directional Mendelian randomization, summary-data-based Mendelian randomization, genome-wide cross-trait analysis

## Abstract

**Background and objectives:**

Observational studies have established a connection between body mass index (BMI) and an increased risk of cognitive decline. However, a comprehensive investigation into the causal relationships between BMI and cognitive function across diverse age groups, as well as the genetic underpinnings of this relationship, has been notably lacking. This study aims to investigate causality and the shared genetic underpinnings of between BMI and cognitive function by conducting a thorough genome-wide analysis, thereby provide valuable insights for developing personalized intervention strategies to promote cognitive health.

**Methods:**

Genetic associations between BMI and cognitive function were thoroughly investigated through covariate genetic analysis and chained imbalance score regression, utilizing data from genome-wide association studies (GWAS). Bi-directional Mendelian Randomization (MR) was employed to uncover associations and potential functional genes were further scrutinized through Cross-trait meta-analysis and Summary-data-based MR (SMR). Subsequently, a detailed examination of the expression profiles of the identified risk SNPs in tissues and cells was conducted.

**Results:**

The study found a significant negative correlation between BMI and cognitive function (β = −0.16, *P* = 1.76E-05), suggesting a causal linkage where higher BMI values were predictive of cognitive impairment. We identified 5 genetic loci (rs6809216, rs7187776, rs11713193, rs13096480, and rs13107325) between BMI and cognitive function by cross-trait meta-analysis and 5 gene-tissue pairs were identified by SMR analysis. Moreover, two novel risk genes *TUFM* and *MST1R* were shared by both cross-trait analysis and SMR analysis, which had not been observed in previous studies. Furthermore, significant enrichment of single nucleotide polymorphisms (SNPs) at tissue- and cell-specific levels was identified for both BMI and cognitive function, predominantly within the brain.

**Conclusion:**

This study uncovers a causal relationship between BMI and cognitive function, with the discovery of *TUFM* and *MST1R* as shared genetic factors associated with both conditions. This novel finding offers new insights into the development of preventative strategies for cognitive decline in obese individuals, and further enhances our understanding of the underlying pathophysiology of these conditions. Furthermore, these findings could serve as a guide for the development of innovative therapeutic approaches to address cognitive decline in obese individuals.

## 1 Introduction

The global population is experiencing an demographic shift towards older age groups, prompting a heightened concern for cognitive decline among the elderly population (Alberini and Chen, 2012). Dementia and mild cognitive impairment (MCI) are among the most diagnosed forms of cognitive impairment (Biessels and Despa, 2018). Estimates suggest that there were approximately 47 million individuals living with dementia worldwide in 2015, a number that is projected to triple by 2050 (Livingston et al., 2017). This anticipated growth will impose a substantial financial and healthcare burden on individuals, their families, and public health services (Petersson and Philippou, 2016). Moreover, the World Health Organization reports that the prevalence of obesity, defined as a body mass index (BMI) of 30 kg/m^2^ or higher, has nearly tripled globally since 1975 (Cirulli et al., 2019), which also presents a substantial public health challenge. Therefore, the population aging and obesity crisis have been two major concerns for public health organizations worldwide.

Previous studies have suggested a causal relationship between BMI and cognitive function; however, the conclusions drawn from these studies have been inconsistent, particularly when examining different age populations. In one hand, some studies have indicated a correlation between higher BMI and a potentially reduced risk of cognitive decline among older adults across various ethnic groups, suggesting that overweight older adults might have a lower propensity for cognitive impairment (Parker et al., 2018; Schmeidler et al., 2019; Wu et al., 2021; Dong et al., 2023; Nicolas et al., 2024). For instance, a community-based prospective cohort study revealed that overweight older adults had a decreased risk of cognitive impairment, whereas significant weight loss was linked to an elevated risk of cognitive decline (Wu et al., 2021). Moreover, two more studies have demonstrated that being underweight is associated with an increased risk of cognitive impairment in older adults (Dong et al., 2023; Nicolas et al., 2024). In the other hand, higher BMI was found to be associated with cognitive decline in younger and mid-life populations (Meo et al., 2019; West et al., 2021; Albanese et al., 2012; Li et al., 2021; Dahl et al., 2013; Smith et al., 2022). For instance, the research conducted by Albanese et al. revealed that an increase in BMI between the ages of 26 and 36 is correlated with lower memory scores (Albanese et al., 2012). Li et al. discovered that obesity is linked to an elevated risk of dementia in the 40–49-year age populations (Li et al., 2021). Another investigation involving individuals aged 50–64 years showed a significant association between BMI and an increased likelihood of MCI (Smith et al., 2022). Therefore, the relationship between BMI and cognitive function remains elusive across diverse age groups.

Importantly, the interplay between BMI and cognitive function has been revealed as bidirectional. One study indicates that a higher BMI adversely affects cognitive function in middle age and beyond, while individuals with better cognitive function tend to maintain a stable weight and avoid weight loss (Karlsson et al., 2021). Additionally, research has identified that patients experiencing cognitive dysfunction often undergo significant weight loss, which is related to the degree of cognitive dysfunction. Notably, this weight loss phenomenon appears to be more pronounced in women than in men (Wirth et al., 2007). However, a study by Norris T et al. found no association between cognitive dysfunction and BMI (Norris et al., 2023). Despite these findings, the existing literature presents limited evidence regarding the reverse causal relationship between BMI and cognitive function, thus leaving uncertainty about the mutual influence between the two. Furthermore, the contribution of genetic factors in determining the overlapping risk profiles of BMI and cognitive function is still largely unexamined. In an effort to fill this gap in our knowledge, we have undertaken an extensive genome-wide analysis to explore these intricate connections.

The hypothesis of the study is that there is a causal relationship between BMI and cognitive function, which is likely mediated by shared genetic factors. To explore this connection, this research focused on investigating the possible causal pathways and genetic associations linking BMI and cognitive function by conducting a thorough analysis of an extensive genome-wide association study (GWAS) dataset. We initiated our study by leveraging genetic correlation and local genetic correlation methodologies to evaluate the extent of shared genetic influences on cognitive function and BMI. To further refine our understanding of specific genetic associations, we engaged in cross-trait meta-analyses. Moving forward, we employed a bidirectional two-sample Mendelian Randomization (MR) analysis to investigate any potential causal relationships between the two traits. In the final analysis, we utilized the genotype-tissue expression (GTEx) and single-cell RNA sequence (scRNA) databases to delve into tissue-specific and cell-type-specific functions. This allowed us to explore the potential genetic pathways that might connect cognitive function and BMI. Ultimately, this comprehensive approach would shed light on the intricate interplay between BMI and cognitive function, providing critical insights into the relationship between these two vital health indicators.

## 2 Materials and methods

### 2.1 Study design, data summary and quality control

**FIGURE 1:**
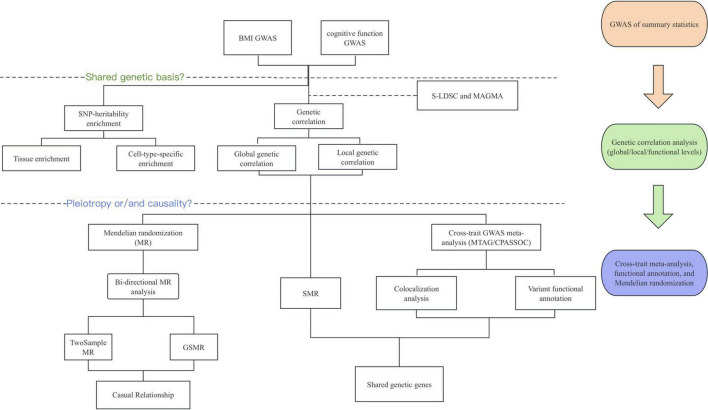
Overview of the experimental design. The genome-wide cross-trait analysis and bi-directional Mendelian randomization were used for identifying shared genetic etiology and causality between BMI and cognitive function. BMI, body mass index; GWAS, Genome-wide Association Study; S-LDSC, Stratified Linkage Disequilibrium Score Regression; MAGMA, Multi-marker Analysis of GenoMic Annotation; MR, Mendelian Randomization; SMR, Summary-databased Mendelian Randomization; GSMR, Generalized Summary-data-based Mendelian Randomization; MTAG, multi-trait analysis of GWAS; CPASSOC, Cross-phenotype association analysis.

The overall study design is illustrated in Figure 1. We extracted summary statistical data for BMI (n = 68,1275) and cognitive function (n = 257,841) from the MRC Integrative Epidemiology Unit OpenGWAS database, accessible at https://gwas.mrcieu.ac.uk/.

The BMI data utilized in this investigation were sourced from the GWAS meta-analysis conducted by the Genetic Investigation of Anthropometric Traits (GIANT) Consortium. This Consortium’s dataset provided impact estimates for BMI-associated single nucleotide polymorphisms (SNPs), which were derived by integrating participants’ weight and height information (Yengo et al., 2018). The Body Mass Index (BMI) values in this study spanned a range from 13.65 to 50.41 kg/m^2^, with an average of 24.89 ± 3.81 kg/m^2^.

The GWAS summary statistics for cognitive function utilized in this investigation were sourced from the MRC Integrative Epidemiology Unit’s OpenGWAS database. These data were obtained through a meta-analysis of several cognitive function-related metrics, including self-reported math ability (N = 564,698 participants), highest math ability (N = 430,445 participants), cognitive ability (N = 35,298 participants), and cognitive performance (N = 222,543 participants), as reported by Lee et al (Lee et al., 2018). The GWAS for self-reported math ability and highest math ability phenotype was carried out exclusively with participants from the personal genomics company 23andMe, who provided survey responses detailing their mathematical proficiency. Cognitive ability data were extracted from published research on general cognitive ability, conducted by the COGENT Consortium, and cognitive performance data was gathered from genome-wide association analyses of cognitive performance by the UK Biobank. Participants ranged in age from 39 to 97 years, with an average age of 64 ± 7 years. This is documented in more detail in Supplementary Table 1.

### 2.2 Heritability and genetic correlation

We used linkage disequilibrium score regression (LDSC) as a valuable method for estimating genetic correlations between multiple traits or diseases (Ni et al., 2018). In order to achieve this goal, we made use of pre-calculated linkage disequilibrium (LD) scores obtained from the 1000 Genomes Project. Additionally, we calculated SNPs using the HapMap 3 SNP set and eliminated SNPs that did not align with the reference panel. Using GWAS summary statistics and LD scores from the 1000 Genomes Project’s European Ancestry Reference Data, we applied LDSC to calculate the heritability of an individual trait and to determine the genetic strength of two genetic correlations between traits. Genome-wide genetic correlations (rg) quantify the average shared genetic effect between two traits that are not affected by environmental confounders, with estimates ranging from −1 (negative correlation) to 1 (positive correlation). Importantly, LDSC can provide accurate estimates even in cases where test statistics may be inflated due to polygenicity.

Our research uses stratified LDSC (S-LDSC) to analyze the impacts of different genomic functional components in order to shed light on the genetic connections between cognition and BMI, which functions by categorizing SNPs into different functional groups and then calculating LD scores for each SNP within a specific category (Gazal et al., 2019). These LD scores are then used to estimate genetic correlations within specific functional categories. To further investigate the functions of genomic components, we conducted a comprehensive LDSC analysis to explore the genetic correlations in a hierarchical manner. We considered several functional categories including coding regions, conserved regions, DNA zyme digital genomic imprinted regions (DGFs), DNA zyme I hypersensitive sites (DHSs), fetal DHSs, intronic regions, promoters, repressor regions, super-enhancers, transcribed regions, and histone marks (Mize and Evans, 2022). By using this approach, we were able to calculate the genetic correlations for each of the different functional categories, thereby revealing the influence of several genomic components on the overall genetic correlation between cognitive function and BMI.

### 2.3 Local genetic correlation analysis

Summary statistical heritability estimation (ρ-HESS) (Shi et al., 2017) was used to estimate local SNP heritability and genetic correlation. ρ-HESS allows us to determine whether BMI and cognitive function are linked genetically in distinct, locally independent regions of the genome. This approach identified 1,703 potential regions that are approximately non-LD related, with an average size of nearly 1.5 MB. We then utilized the 1000 Genomes Project as a reference, as suggested on the ρ-HESS webpage, to ascertain the genetic correlation between BMI and cognitive function and to estimate the local SNP heritability of these traits. Subsequently, to manage the risks associated with multiple testing, we applied the Bonferroni correction, adjusting the significance threshold to account for the number of regions under investigation.

### 2.4 Cross-trait GWAS meta-analysis

To identify common risk SNPs associated with both BMI and cognitive function, we employed two cross-trait meta-analyses: the multi-trait analysis of GWAS (MTAG) (Turley et al., 2018) and the Cross-Phenotype Association analysis (CPASSOC) (Liu et al., 2021). MTAG benefits from incorporating a diverse array of related traits, which enhances the accuracy of SNP effect size estimations for each trait. MTAG is applicable when the SNP heritability is assumed to be equal across traits and when genetic covariance between traits is fully characterized. We determined the upper bound of the error discovery rate using “maxFDR.” Furthermore, we employed the CPASSOC to conduct a pairwise cross-trait meta-analysis, considering the varying heritability estimates across the two CPASSOC traits. This approach involved calculating the Statistical Heterogeneity (SHet) and P-values between traits through a meta-analysis of GWAS aggregated data. It incorporates the inter-trait effect diversity, utilizing sample size weights to appropriately account for it. This method is designed to address the varying effects among traits, offering an advantage over the SHom (fixed effects meta-analysis method), which is less effective at handling such heterogeneous effects. SHet has been shown to provide superior and more robust statistical power. For our analysis, we utilized SHet to integrate summary data for cognitive function and BMI.

Relevant SNPs were those demonstrating a strong association with both BMI and cognitive function (P < 5 x 10^−8 in both MTAG and CPASSOC). To combine the summary statistics for BMI and cognitive function, we utilized the SHet method, which accounts for statistical heterogeneity. This was followed by clumping in PLINK (version 1.9) to identify independent SNPs most significantly associated with the phenotype. The PLINK “clumping” function was applied with the following parameters: -clump-p1 5e-8 -clump-p2 1e-5 -clump-r2 0.2 -clump-kb 500. Novel loci were defined as independent SNPs that were not in LD with significant SNPs, as determined by the cross-trait GWAS meta-analysis. Specifically, we considered SNPs that were not in LD (r2 > 0.2) within a 1000 kb window with significant SNPs from the cross-trait GWAS meta-analysis, which included the two GWAS studies on BMI and cognitive function conducted in this research.

### 2.5 SNP Annotation

To identify significant and lead SNPs, we annotated the MTAG summary data using the online platform functional mapping and annotation (FUMA).^1^ SNPs were considered to be independent when *P* < 5x10−8 and r2 < 0.6. Notably, lead SNPs, which are characterized by an r2 value of less than 0.1, indicate distinct genetic variants. We employed the default parameters for the Functional Mapping and Annotation within FUMA and referenced the 1000 Genomes Project Phase 3 data, specifically using the European ancestry population as our reference panel. This approach was designed to capitalize on our European heritage as a starting point for annotation. To elucidate tissue-specific associations, we conducted a tissue enrichment analysis, leveraging the GENE2FUNC process within FUMA. This analysis utilized data from 54 tissue types, as provided by the Genotype-Tissue Expression project, version 8 (GTEx v8).

### 2.6 Colocalization analysis

To identify the common genetic basis of cross traits, it is also possible to examine if two GWAS signals are due to separate genetic variants that are close to one another or to the same variants. We employed the coloc package (Wu et al., 2019) to conduct a thorough colocalization examination. Five mutually exclusive hypotheses are identified by Colco: H0 (no association), H1 or H2 (relationship to one trait alone), H3 (association to both traits, two different SNPs), and H4 (connection to both characteristics, one shared SNP). Utilizing a Bayesian algorithm, coloc calculates the posterior probabilities for each of these hypotheses. We received summarized information regarding changes within a 1.0 Mb radius of the index SNP for every shared locus and calculated the posterior probability for H4 (PPH4). At these shared loci, we also retrieved summary statistics for variants located less than 0 Mb from the index SNP and determined the posterior probabilities for both H4 (PPH4) (Lin et al., 2023) and H3 (PPH3). A locus was deemed colocalized if either the PPH4 or PPH3 value exceeded 0.95.

### 2.7 Mendelian randomization analysis

We employed five prominent MR methods—MR-Egger (Bowden et al., 2017), inverse variance weighting (IVW) (Peng et al., 2022), weighted median, weighted model, and Mendelian randomization analysis based on generalized aggregated data (GSMR) (Zhu et al., 2018)—to conduct multiple hypothesis tests for horizontal pluripotency in relation to the causal connection between body mass index BMI and cognitive function. The R software packages “TwoSampleMR” and “GSMR” were utilized to identify any potentially significant associations (P < 0.05). The Wald test was applied to assess the causative link between a single genetic variable and the exposure-outcome relationship. The IVW method facilitated the estimation of causal effects for both phenotypes through meta-analysis. The MR-Egger technique leveraged weighted linear regression to elucidate the presence and direction of unmeasured horizontal pleiotropy, providing insights into potential confounding factors. Cochran’s Q statistic and the MR-Egger intercept test were conducted to evaluate heterogeneity and pleiotropy. To detect pleiotropy and multivalence, as well as potential outliers, we utilized MR-PRESSO analysis and single SNP effect analysis.

The selection of variants for our analysis was guided by three key assumptions: (1) the variants must be associated with the exposure variable; (2) they should not be influenced by confounding factors; and (3) they should not directly affect the outcome variable. To ensure the validity of these assumptions, we focused on identifying SNPs that exhibited genome-wide significance for the exposure traits, with a stringent threshold of *P* ≤ 5 × 10^−8, serving as instrumental variables. The F statistic for each SNP instrument was calculated using the formula F = [(N−k−1)/k] * [R^2/(1−R^2)], where N represents the total number of individuals, k is the number of SNPs, and R^2 is the variance explained by the instrumental variable. SNPs with an F statistic below 10 were considered to provide insufficient information for meaningful analysis and were accordingly excluded. To assess the heterogeneity across studies, the Cochran’s Q statistic was computed. Additionally, sensitivity analyses were conducted using a leave-one-out method to evaluate the robustness of our findings. This involved removing one study at a time and re-calculating the statistics to ensure that the results were not driven by any single outlier.

### 2.8 Tissue specific enrichment of SNP heritability

#### 2.8.1 Large-scale data- sharing consortium (LDSC) analysis

To determine the tissues most significantly associated with shared genes, we conducted a GTEx (Genotype-Tissue Expression) tissue enrichment analysis utilizing the S-LDSC (Sparse Linear Discriminant Analysis with an Enhanced R Package) method (“Human genomics. The Genotype-Tissue Expression (GTEx) pilot analysis: multitissue gene regulation in humans” GTEx Consortium, 2015; Battle et al., 2017). GTEx version 8 provides comprehensive data on 53 distinct tissue types. S-LDSC was applied to evaluate the enrichment of SNP heritability for cognitive function and BMI across various tissues. We analyzed the z-fraction p-value of the regression coefficient to modify the initial model and the entire gene set, assessing the significance of each SNP’s genetic effect richness estimate. Subsequent tissue-specific analyses estimated the associations between genes specifically expressed in each tissue and their impact on cognitive function and BMI. To account for the multiple testing involved, we employed the Bonferroni correction algorithm, setting the significance threshold at 0.05 divided by the number of tissue types (0.05/53).

#### 2.8.2 Multi-marker analysis of genomic annotation (MAGMA) analysis

As a sensitivity analysis complementing the S-LDSC results, we utilized MAGMA (de Leeuw et al., 2015) to conduct tissue-specific enrichment and gene set enrichment analyses. This involved performing gene-level association studies using GWAS pooled data. MAGMA estimates gene-phenotype associations by aggregating the *P* of SNPs in close proximity to the target genes.

### 2.9 Mendelian randomization (MR) based on aggregated data

Summary-data-based MR (SMR) was used to identify potentially statistically significant functional genetic variants that may be associated between cognitive function and BMI. identify functional genes that may be statistically associated between cognitive function and BMI. SMR is an approach that integrates summary statistics from GWAS and expression quantitative trait loci (eQTL) studies within an instrumental variable framework to detect associations between gene expression and target phenotypes (Bowden et al., 2017; Krishnamoorthy et al., 2023). SMR was performed in tissues significantly enriched for cognitive function and BMI SNP heritability. Genome-wide significant SNPs were used as instrumental variables to perform an instrument-dependent heterogeneity (HEIDI) test to assess the presence or absence of a causal link in the observed associations (Wang et al., 2021; Shadrina et al., 2020). An additional step involved using the HEIDI-outlier test to differentiate between causality or multiplicity and linkage. Significantly shared functional genes between cognitive function and BMI were defined as those genes that passed a Benjamini-Hochberg false discovery rate (FDR) test and a HEIDI outlier test for functional genes with a significance level of *P* < 0.05, with a requirement of 10 SNPs for SMR analysis for both traits.

### 2.10 Cell-type enrichment analysis using scRNA-seq data

We performed MAGMA cell typing using scRNA-seq (Zhang et al., 2023) datasets to assess cell type expression-specific gene height genetic correlations between BMI and cognitive function. For this purpose, we calculated regression coefficient z-scores for all genes. Subsequently, we used functional localization and annotation (FUMA) processing of SNP genes on GTEx tissue to assess the significance of each gene’s heritability enrichment estimate. Only scRNA-seq datasets of tissues enriched for both cognitive function and BMI were included in the cell-type specific analysis. We used the Bonferroni program to correct for multiple testing, and only significantly correlated cell types were identified by correcting the average gene expression in the preprocessed scRNA-seq dataset and combining it with the MAGMA results for each trait.

## 3 Results

### 3.1 Genetic correlations

We included a comprehensive dataset consisting of 2,336,260 SNPs associated with cognitive function and 10,066,414 SNPs related to BMI (Supplementary Table 2). The single trait LDSC s analysis revealed GWAS-based estimates of heritability for cognitive function (GWAS_cognitive function_) to be 0.2002 (SE = 0.007) and for BMI (GWAS_BMI_) to be 0.2126 (SE = 0.0069), with mean statistics of 2.0299 and 3.9461 for GWAS_cognitive function_ and GWAS_BMI_, respectively. Pairwise LDSC analysis indicated a genome-wide negative correlation between BMI and cognitive function (rg = −0.1237, SE = 0.0151, *P*-LDSC = 3.1453 x 10^−16), as presented in Supplementary Table 3. The genetic covariance intercept was estimated to be approximately 0.01. Considering the minor overlap in sample sets, we conducted a constrained-intercept LDSC analysis without assuming overall stratification, which showed a slightly weaker but still statistically significant genetic correlation (Supplementary Table 3).

### 3.2 Local genetic correlation analysis

We conducted a genome-wide analysis to assess localized genetic correlations between BMI and cognitive function, utilizing summary statistics-based heritability estimates. Following multiple correction procedures, we identified strong genetic correlations in 18 distinct regions that emerged as the most significant (Supplementary Figure 1 and Supplementary Table 4).

### 3.3 Partitioned genetic analysis

In our study, SNPs linked to BMI were found to be enriched in 48 out of 97 functional categories, with the most significant enrichment observed in the MAF_Adj_ASMCL2_0 category (Enrichment = 1.94025 x 10^13, *P* = 7.51 x 10^−24). Similarly, SNPs associated with cognitive function showed enrichment in 48 out of 97 functional categories, with the MAF_Adj_ASMCL2_0 category again displaying the highest level of enrichment (Enrichment = 2.24 x 10^13, *P* = 8.55 x 10^−25) (Supplementary Tables 5–6). Additionally, 39 functional regions were significantly enriched for both BMI and cognitive function, as shown in Supplementary Table 7.

### 3.4 Causal relationship between BMI and cognitive function

**FIGURE 2:**
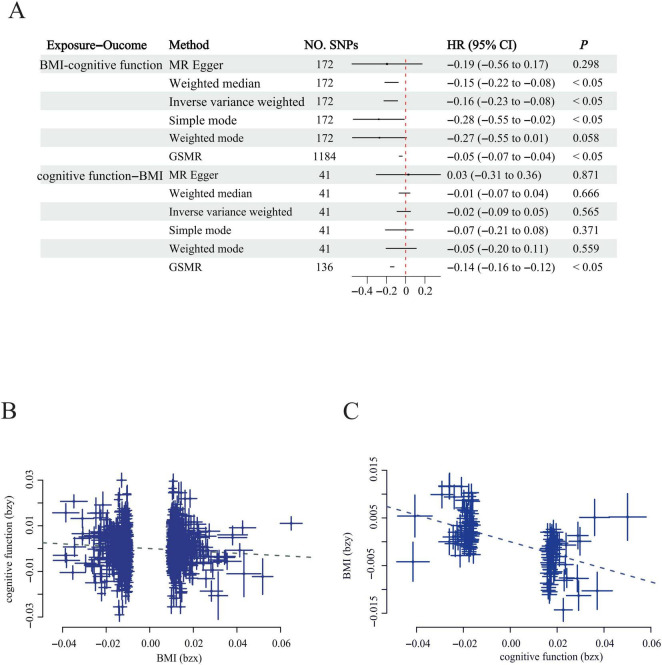
Bi-directional MR analysis and GSMR effect size plots for the relationship Between cognitive Function and BMI. **(A)** Bi-directional MR Forest Plot: The upper panel illustrates the causal effect of cognitive function on BMI, while the lower panel depicts the causal effect of BMI on cognitive function; **(B)** GSMR effect size plot for the impact of BMI on cognitive function; **(C)** GSMR effect size plot for the impact of cognitive function on BMI. Each dot corresponds to a SNP. The effect estimates and their 95% confidence intervals (CI) are shown with squares and whiskers, respectively. BMI: body mass index; SNP: single-nucleotide polymorphism.

We conducted bi-directional MR analyses to delineate the causal relationships between the variables of interest and to ascertain whether the underlying genetic architecture exhibited heterogeneity and pleiotropy. Instrumental variables (IVs) were chosen in accordance with three a priori hypotheses (Supplementary Table 8), and subsequent using a bi-directional MR framework was employed to assess the consistency and stability of the inferred causal associations. Our investigation encompassed five distinct MR methodologies, which collectively provided evidence supporting a causal link between BMI and cognitive function (IVW β = −0.16, *P* < 0.05; GSMR β = −0.05, *P* < 0.05), as depicted in Figure 2, Supplementary Figure 2, and Supplementary Table 8, with negligible indication of substantial heterogeneity or pleiotropy. In the converse causal direction, examining the influence of cognitive function on BMI, all employed MR techniques except for GSMR failed to reach statistical significance (GSMR β = −0.05, *P* < 0.05), as illustrated in Figure 2.

### 3.5 Cross-trait meta-analysis

**FIGURE 3:**
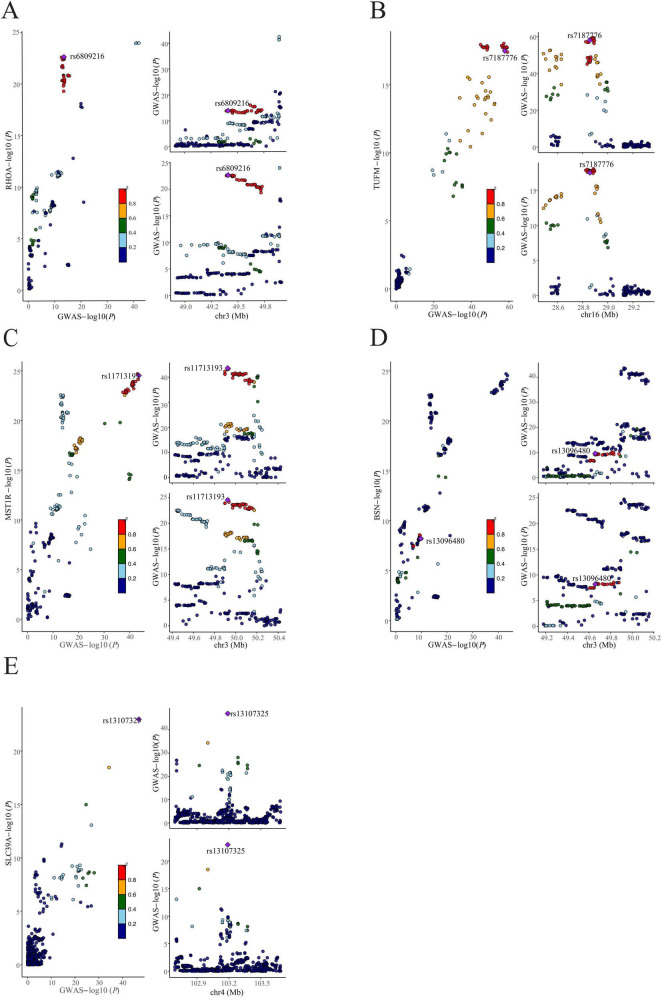
Colocalization analysis of shared SNPs between BMI and cognitive function. **(A)** rs6809216, **(B)** rs7187776, **(C)** rs11713193, **(D)** rs13096480, and **(E)** rs13107325 are five identified shared SNPs between BMI and cognitive function. In each panel, the diamond-shaped purple points indicate the SNPs that exhibit the minimal sum of *P*-value in corresponded BMI and cognitive function.

Drawing on genetic correlations and causal outcome effects, there is evidence of a genetic mechanism underlying the relationship between BMI and cognitive function. Utilizing the MTAG methodology, we conducted an analysis of GWAS_BMI_ and GWAS_cognitive function_, identifying 1141 SNPs that exhibited genome-wide significance (*P* < 5 x 10^**−**8). To corroborate the findings in the MTAG, we employed CPASSOC for multiplicity analysis, integrating the results of both MTAG and CPASSOC to refine the selection of the 1141 SNPs (*P* < 5 x 10^**−**8). Subsequently, we used PLINK to extract 44 SNPs from the CPASSOC results. Furthermore, by consolidating the SNPs from the ρ-HESS significant regions with the novel SNPs, we identified seven novel SNPs, which are associated with the combined phenotype of BMI-cognitive function (Table 1). Furthermore, co-localization analysis revealed that five of these SNPs including rs6809216, rs7187776, rs11713193, rs13096480, and rs13107325, demonstrate co-localization between BMI and cognitive function (PH4 > 0.95) (Figure 3 and Table 2).

**TABLE 1:** Pleiotropic loci between BMI and cognitive function.

SNP	CHR	Position	GENE	A1	A2	MTAG Beta1	MTAG SE1	MTAG *P*1	MTAG Beta2	MTAG SE2	MTAG *P*2	*P*_Shet	*P*_Shom
rs2196172	2	198889893	PLCL1	G	T	0.01111566	0.001707339	7.49E-11	−0.015337213	0.002773543	3.21E-08	1.59E-14	0.001737162
rs6809216	3	49412559	RHOA	A	G	−0.0147 42048	0.001864157	2.61E-15	0.030812745	0.002987125	6.02E-25	4.23E-27	0.063307014
rs13096480	3	49658084	BSN	G	A	−0.0148 36765	0.002221177	2.39E-11	0.021547338	0.003504589	7.83E-10	6.94E-16	0.003113093
rs11713193	3	49924424	MST1R	A	G	0.024502432	0.001705763	8.64E-47	−0.030771579	0.002768231	1.05E-28	5.23E-65	6.52E-14
rs13107325	4	103188709	SLC39A8	T	C	0.045625487	0.00305112	1.47E-50	−0.051900093	0.004820966	5.01E-27	2.51E-68	1.64E-16
rs7124681	11	47529947	CELF1	A	C	0.02672277	0.001615749	1.92E-61	−0.01819543	0.002783373	6.27E-11	2.48E-67	3.02E-33
rs7187776	16	28857645	TUFM	G	A	0.027206499	0.00162231	4.03E-63	−0.027717531	0.002903956	1.36E-21	7.51E-78	2.68E-26

CHR, chromosome; SNP, single-nucleotide polymorphism; MTAG, multi-trait analysis of genome-wide association studies; SE, standard error; 1: BMI, 2: cognitive function.

**TABLE 2:** Posterior probability test of shared loci between BMI and cognitive function.

SNP	GENE	PPH4
rs2196172	PLCL1	0.050834901762781
**rs6809216**	**RHOA**	**0.997655251941814**
**rs13096480**	**BSN**	**0.991551932510143**
**rs11713193**	**MST1R**	**0.991374521631222**
**rs13107325**	**SLC39A8**	**0.999999956976809**
rs7124681	CELF1	0.708825902651126
**rs7187776**	**TUFM**	**0.95629072739858**

SNP, single-nucleotide polymorphism; PPH4, the posterior probability of hypothesis 4. Bold values: colocalized SNPs.

### 3.6 Summary statistics-based Mendelian randomization (SMR)

**FIGURE 4:**
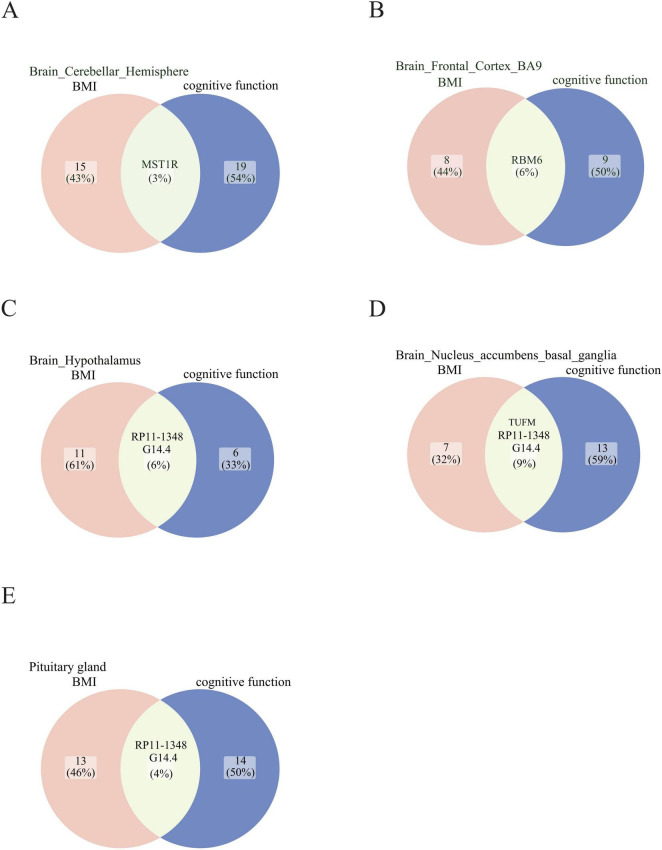
Summary statistics-based Mendelian Randomization (SMR) analysis based on aggregated data. Candidate genes that exhibit shared associations with BMI and cognitive function are found in **(A)** Brain cerebellar hemisphere, **(B)** Brain frontal cortex BA9, **(C)** Brain hypothalamus, **(D)** Brain nucleus accumbens basal ganglia, and **(E)** Pituitary gland. The shade of green indicates the number of genes shared between the BMI and cognitive function traits in each brain region.

Through the concurrent analysis of GWAS data from the GETx consortium and eQTLGen, we employed the SMR approach to infer the causal relationship between BMI and cognitive function, as well as to identify putative functional genes that passed the HEIDI outlier test. Our investigation uncovered genes that exhibit shared associations with BMI and cognitive function in the cerebellar hemisphere, frontal cortex, hypothalamus, basal ganglia, and pituitary gland (Figure 4 and Table 3). Notably, *TUFM* (*P*SMR = 4.94E-11, *P*HEIDI = 0.32) and *MST1R* (*P*SMR = 8.15E-11, *P*HEIDI = 0.06) were identified as two novel significant candidates by cross-trait analysis.

**TABLE 3:** Shared significant SMR associations for BMI and cognitive function in enriched tissues.

Gene	Probe ID_BMI	CHR	Probe_ position	Effect allele	Another allele	Beta_ SMR	SE_SMR	*P*_ SMR	*P*_ HEIDI	No. SNP _HEIDI
**Brain_Cerebellar_Hemisphere**										
MST1R	ENSG00000164078	3	49924435	C	T	0.0484738	0.00746002	8.15E-11	0.06277367	13
**Brain_Frontal_Cortex_BA9**										
RBM6	ENSG00000004534	3	49977440	T	C	−0.0562046	0.00700223	1.00E-15	0.1334418	18
**Brain_Hypothalamus**										
RP11-1348G14.4	ENSG00000251417	16	28814097	C	T	−0.0394032	0.00688223	1.03E-08	0.1583842	12
**Brain_Nucleus_accumbens_basal_ganglia**										
TUFM	ENSG00000178952	16	28853732	C	T	0.0907204	0.0138028	4.94E-11	0.3168625	13
RP11-1348G14.4	ENSG00000251417	16	28814097	C	T	−0.0459571	0.0077102	2.51E-09	0.3574173	13
**Pituitary gland**										
RP11-1348G14.4	ENSG00000251417	16	28814097	T	C	−0.0554573	0.0076976	5.83E-13	0.05697258	18

CHR, chromosome; SNP, single-nucleotide polymorphism; SE, standard error; 1: BMI; 2: cognitive function.

### 3.7 LDSC-SEG and MAGMA tissue enrichment

Utilizing publicly available GWAS data, which were enhanced with SNP GTEx data, we applied LDSC Squared Error Gain (LDSC-SEG) method to pinpoint tissues with heightened gene expression due to shared SNPs. By modifying the baseline model, we detected significant SNP heritability enrichment in 53 tissues associated with BMI, with the cerebral frontal cortex showing the most pronounced enrichment (*P* = 0.001). In terms of cognitive function, 49 tissues demonstrated significant enrichment, and the frontal cortex of the brain emerged as the most highly enriched tissue (*P* = 4.83x10^−8; Supplementary Table 9).

To conduct a sensitivity analysis of the S-LDSC method, we used MAGMA, a tool designed to analyze GWAS data. The analysis revealed that BMI-related SNPs were particularly enriched in eight distinct brain regions, with the cerebellum being the predominant region. For cognitive function, we observed specific enrichment of SNPs in 14 different brain regions, again, with the cerebellar hemisphere showing the most significant enrichment (Supplementary Figures 3–4). These findings provide insights into the tissues and brain regions where shared SNPs associated with both BMI and cognitive function are most active, highlighting the complex interplay between genetic factors and these traits. The results underscore the importance of considering both genetic and tissue-specific factors when investigating the etiology of complex traits such as BMI and cognitive function.

### 3.8 Cell-specific SNP heritability enrichment

To further explore the potential role of shared SNPs within different specific cell types, cell-specific SNP heritability enrichment was performed. In this study, the prefrontal cortex, hippocampus, and midbrain are three key brain regions where SNPs associated with both BMI and cognitive function were found to be particularly enriched within neuronal populations (Supplementary Figure 5). First, in the prefrontal cortex, a region of the brain involved in higher cognitive functions such as decision-making, planning, and social behavior, GABAergic neuronal cells play a crucial role in the regulation of both cognitive function and BMI. Second, in the hippocampus, a region of the brain involved in learning and memory, the GABA2 neuronal cells emerges as a potential significant factor contributing to the interplay between BMI and cognitive function. Last, in the midbrain, SNPs related to BMI and cognitive function were significantly enriched in neuronal subsets, with a notable enrichment observed in certain neuron types, including Gaba, NbGaba, NbML5, and DA1. Overall, the findings provide insights into the roles of GABAergic neurons in the regulation of cognitive function and BMI and suggest that genetic variations within these neurons may contribute to the interplay between these traits. Further research is needed to fully understand the mechanisms by which these genetic variations contribute to the regulation of cognitive function and BMI, and to explore the potential implications for the development of interventions to improve cognitive function and reduce the risk of obesity.

## 4 Discussion

In this study, we provide evidence indicating a causal link between BMI and cognitive function. We also emphasize the convergence in the genetic architecture that underpins both BMI and cognitive function. Our research, which are based on results spanning various age cohorts, advances our comprehension of the intertwined complexities associated with these conditions. These insights may prove valuable in enhancing diagnostic accuracy, predicting outcomes, and devising targeted therapeutic interventions for related disorders.

Through the application of cross-trait meta-analysis, including MTAG and the CPASSOC, we identified 44 SNPs that were significantly associated with both BMI and cognitive function. Within these, we pinpointed five loci that reside in highly significant regions: *RHOA, BSN, MST1R, SLC39A8*, and *TUFM*. Notably, *RHOA, BSN*, and *SLC39A8* have previously been implicated in the association between cognitive function and BMI (Xiang et al., 2022; Iyer et al., 2021; Zhu et al., 2023; Liu et al., 2023). Importantly, to mitigate potential errors associated with sample overlap, we employed both MTAG and CPASSOC methods, and further investigated the SNPs discovered by MTAG in relation to CPASSOC risk findings. The convergence of SNPs identified via MTAG with those from CPASSOC analysis enhanced the reliability of our results, reinforcing the validity of our findings.

In addition to the cross-trait meta-analysis, we integrated GWAS data from GTEx and eQTLGen to identify shared risk genes that might underlie the correlation between BMI and cognitive function. Among these shared risk genes, *TUFM* and *MST1R* were identified. Previous research by Lin J et al. has uncovered a novel pathway for the regulation of mitochondrial autophagy, mediated by PINK1 and its kinase substrate TUFM (Lin et al., 2020). *TUFM* has been implicated in Alzheimer’s disease (AD) pathology by regulating BACE1 translation, apoptosis, and Tau phosphorylation (Zhong et al., 2021). Additionally, TUFM has been associated with obesity (Lee et al., 2016; Gutierrez-Aguilar et al., 2012). *MST1R*, on the other hand, has primarily been detected in various tumors, including pancreatic cancer (Babicky et al., 2019), nasopharyngeal carcinoma (Dai et al., 2016), prostate cancer (Batth et al., 2021), and breast cancer (Millar et al., 2020). By the cross-trait analysis and SMR analysis, *TUFM* and *MST1R* are identified to have a potential relationship with both BMI and cognitive function, which indicates a novel perspective on the shared genetic mechanisms that could contribute to the co-occurrence of these conditions. This provides a novel direction for future prevention and intervention strategies targeting cognitive function decline in obese individuals.

In this study, MR analysis have provided a definitive causal association between BMI and cognitive function, suggesting a positive correlation (Mina et al., 2023). Our investigation revealed a causal effect of BMI on cognitive function, with an inverse relationship between BMI increase and the risk of cognitive impairment onset, thus positioning BMI as a protective factor—aligning with earlier research findings (Veronese et al., 2017; Siervo et al., 2011; Grapsa et al., 2023; Aiken-Morgan et al., 2020). Although we observed some correlation in GSMR analysis in the opposite direction, we are unable to ascertain at this juncture whether this relationship holds significance. These findings imply that individuals with cognitive impairment might benefit from increasing their BMI to mitigate the risk of disease progression. However, the potential for BMI alterations among those already experiencing cognitive impairment needs to be further explored. Moreover, to evaluate the validity of our results, we adopted multiple MR techniques in conjunction with standard MR methodologies. The lower and weighted median approaches were found to provide smaller effect estimates yet higher type I error rates in comparison to the IVW method. Conversely, MR-Egger regression produced less biased estimates across the board. The GSMR method, which employs a generalized least squares strategy for estimation, differs from the IVW approach. Given that the causal relationship between BMI and cognitive function remained significant across all three analytical methods, the observed effects are considered plausible within the context of this study.

Utilizing the GTEx dataset, we conducted an extensive analysis of the tissue-specific SNP genetic power enrichment concerning the relationship between BMI and cognitive function via the S-LDSC and MAGMA methods. Our investigation revealed eight prevalent regions of enrichment, with the cerebral cortex emerging as the predominant site. The suggestion is that BMI-related alterations in the brain may precipitate common lesion areas, potentially influencing brain structural changes that are linked to BMI (Willeumier et al., 2011; Opel et al., 2021; Medawar and Witte, 2022). Furthermore, evidence suggests that an overweight BMI alters brain chemistry, thereby impacting cognitive function (Xu et al., 2023; Asch et al., 2022). These findings underscore a shared pathophysiological framework involving brain anatomy and BMI in the realm of cognitive function. In particular, our study highlighted significant enrichment for BMI and cognitive function in the hippocampus, midbrain, and cerebral cortex. Previous research has indicated that GABA neurons in the hippocampus are integral to synaptic plasticity (He and Bausch, 2014), while chronic activation of GABA cells in the midbrain has been shown to ameliorate obesity induced by a high-fat diet in mice (Wang et al., 2023). Conversely, inhibition of GABAergic transmission in the cerebral cortex and hippocampus is associated with cognitive impairments (Bast et al., 2017). Additionally, the loss of dopamine neurons within the substantia nigra pars compacta is a hallmark feature of Parkinson’s disease (PD) (Kamath et al., 2022). The influence of leptin on GABA neurons in the cerebral cortex has also been implicated in the prevention of obesity (Vong et al., 2011). The role of NbML5 cells in the midbrain in the context of BMI and cognitive function needs further investigation to elucidate its mechanisms.

The present study offers several notable strengths. Firstly, e meticulously identified genes linked with both BMI and cognitive function through a rigorous cross-trait analysis and SMR results. This approach significantly enhanced the methodological robustness of our investigation. Secondly, to further ascertain the causal connection between BMI and cognitive function, we incorporated GSMR into our analytical framework, as opposed to the previous single MR approach to reinforce the reliability of our findings. Lastly, we conducted detailed tissue and cellular enrichment analyses for both conditions, providing a comprehensive elucidation of the interplay between BMI and cognitive function at the tissue-cell-gene level.

Meanwhile, we acknowledge the inherent limitations of our research. Firstly, our findings are primarily drawn from European populations, underscoring the need for future studies to broaden their scope to encompass other ancestral groups. This expansion is crucial for a comprehensive understanding of the biological mechanisms governing the relationship between the two traits. Secondly, due to the absence of detailed demographic information on our study population, we were unable to assess disease risk disparities across genders and ethnicities. Moreover, our investigation did not explore the etiology of cognitive function, indicating a requirement for larger GWAS datasets focused on both BMI and cognitive function to corroborate our findings across different ethnicities. Lastly, we did not explore whether similar associations exist between BMI and neurodevelopmental disorders within younger populations, despite our results suggest a causal link between BMI and cognitive function across various age groups. Future studies should explore this potential connection, as current literature suggests that children and adolescents with autism spectrum disorder often exhibit higher BMI (Sammels et al., 2022), altered prefrontal cortex GABA balance (Maier et al., 2022), cognitive difficulties (Masedu et al., 2021), and challenges in social functioning (Pino et al., 2020). It would be interesting to explore them as well. Exploring these inter relationships could provide valuable insights into the complex interplay between BMI and neurodevelopmental conditions.

In summary, this is a comprehensive and systematic study applying genome-wide association data to explore the causal relationship and shared genetic architecture between BMI and cognitive function. Our research leverages extensive GWAS datasets and tissue-specific cell type expression data to present new evidence suggesting a genetic correlation between BMI and cognitive function.

## 5 Conclusion

In conclusion, this study has unveiled a negative correlation between BMI and cognitive function across different age populations, identifying *TUFM* and *MST1R* as novel genetic markers that influence both BMI and cognitive function. This critical discovery not only enriches our understanding of the physiological underpinnings of both obesity and cognitive decline, but also offers crucial insights for the development of preventive strategies to counteract cognitive deterioration in individuals with obesity.

## Data Availability

Publicly available datasets were used for analysis in this study. Data about the body mass index can be acquired at: https://gwas.mrcieu.ac.uk/datasets/ieu-b-40/. Data about cognitive function can be acquired at: https://gwas.mrcieu.ac.uk/datasets/ebi-a-GCST006572/.

## Footnotes

1
https://fuma.ctglab.nl/
