# Supplementary material for: Investigating causality and shared genetic architecture between body mass index and cognitive function: a genome-wide cross-trait analysis and bi-directional Mendelian randomization study
Source: Front Aging Neurosci. 2024 Oct 16;16:1466799. doi: 10.3389/fnagi.2024.1466799 (PMC11522962; doi:10.3389/fnagi.2024.1466799)
Supplement: Supplementary file 1 [file Table_1.doc]

Supplementary Material

# Supplementary Tables

**Supplementary Table 1. Phenotype Descriptions and distributions in cognitive function GWAS data**

| Phenotype | Cohort | Phenotype measure(s) |
| --- | --- | --- |
| Math Ability  (Self Report) | 23andMe | How would you rate your mathematical ability?  i. I’m not sure [missing] ii. Very poor [0] iii. Poor [1] iv. About average [2] v. Good [3] vi. Excellent [4] |
| Highest Math Class | 23andMe | Excluding statistics courses, what is the most advanced math class you have successfully completed?   i) Pre-Algebra [1] ii) Algebra [2] iii) Geometry [3] iv) Trigonometry [4] v) Pre-Calculus [5] vi) Calculus [6] vii) Vector Calculus [7] viii) More than vector calculus [8]  ix) I'm not sure [missing] |
| Cognitive ability (COGENT Consortium) | COGENT Consortium | The COGENT Consortium's cognitive performance data are statistically derived broadband indices of interpersonal performance in a neuropsychological test battery. As a COGENT participant, data from at least one neuropsychological measure from at least three cognitive performance domains (e.g., digit breadth of working memory; logical memory in lieu of verbal declarative memory; and processing speed of digit-symbol encoding), or the use of validated g-sensitive measures, are required. Digit symbol encoding, digit span, verbal memory for words, visual memory, word reading, semantic fluency, verbal memory for stories, vocabulary, phonemic fluency, and the trajectory test were the most common tests used in the cohort. An average of 8 (sd ± 4) neuropsychological tests were administered per COGENT substudy. |
| Cognitive performance  (UKB) | UKB | Standardized scores for language-numerical reasoning tests. The test contains 13 logic and reasoning questions with a time limit of two minutes and is designed to measure fluid intelligence. The records include reaction time, pairs matching, and prospective memory. Each respondent took the test up to four times, and we used the average of the standardized scores, which were then standardized. |

**Supplementary Table 2. Details of GWAS summary data**

| Phenotype | **N** | **Ethnicity** | **No. SNPs (Quality Control)** |
| --- | --- | --- | --- |
| Body mass index | 68,1275 | European | 2,336,260 |
| Cognitive function | 257,841 | European | 10,066,414 |

**N:** samplesize.

**Supplementary Table 3. Genetic correlations estimated using linkage disequilibrium score regression (LDSC) with and without constrained intercept**

LDSC test without constrained intercept

Heritability of BMI

---------------------------

Total Observed scale h2: 0.2151 (0.0059)

Lambda GC: 2.7872

Mean Chi^2: 3.9461

Intercept: constrained to 1

Heritability of cognitive function

-----------------------------

Total Observed scale h2: 0.1988 (0.0052)

Lambda GC: 1.7218

Mean Chi^2: 2.0299

Intercept: constrained to 1

Genetic Covariance

------------------

Total Observed scale gencov: -0.0276 (0.0024)

Mean z1*z2: -0.2359

Intercept: constrained to 0

Genetic Correlation

-------------------

Genetic Correlation: -0.1334 (0.0118)

Z-score: -11.3401

*P*: 8.3026E-30

LDSC test with constrained intercept

Heritability of BMI

---------------------------

Total Observed scale h2: 0.2126 (0.0069)

Lambda GC: 2.7872

Mean Chi^2: 3.9461

Intercept: 1.0184 (0.0269)

Ratio: 0.0063 (0.0091)

Heritability of cognitive function

-----------------------------

Total Observed scale h2: 0.2002 (0.007)

Lambda GC: 1.7218

Mean Chi^2: 2.0299

Intercept: 0.9952 (0.0137)

Ratio < 0 (usually indicates GC correction).

Genetic Covariance

------------------

Total Observed scale gencov: -0.0255 (0.0032)

Mean z1*z2: -0.2359

Intercept: -0.0104 (0.0103)

Genetic Correlation

-------------------

Genetic Correlation: -0.1237 (0.0151)

Z-score: -8.1676

*P*: 3.1453E-16

**Supplementary Table 4. Local genetic correlation between BMI and cognitive function**

| **CHR** | **Start position** | **End position** | **No. SNPs** | **K** | **local_rhog** | **Var** | **SE** | **Z-store** | ***P*** |
| --- | --- | --- | --- | --- | --- | --- | --- | --- | --- |
| 1 | 71684405 | 74326907 | 1440 | 50 | 0.00032657 | 3.00E-09 | 5.48E-05 | 5.9623 | 2.49E-09 |
| 1 | 83991748 | 84844495 | 524 | 40 | -0.00013159 | 9.52E-10 | 3.08E-05 | -4.2659 | 1.99E-05 |
| 1 | 111741737 | 113273306 | 1211 | 50 | -0.00020626 | 1.81E-09 | 4.26E-05 | -4.844 | 1.27E-06 |
| 1 | 201589975 | 203334734 | 891 | 50 | -0.00020594 | 2.34E-09 | 4.84E-05 | -4.253 | 2.11E-05 |
| 2 | 24686918 | 26894985 | 917 | 50 | 0.00027879 | 3.21E-09 | 5.67E-05 | 4.9196 | 8.67E-07 |
| 2 | 98995201 | 101822329 | 1757 | 50 | -0.00025148 | 2.49E-09 | 4.99E-05 | -5.0363 | 4.75E-07 |
| 2 | 164466638 | 165178840 | 376 | 27 | -0.00014706 | 1.12E-09 | 3.35E-05 | -4.3863 | 1.15E-05 |
| 2 | 198078110 | 199311125 | 547 | 32 | -0.00016625 | 1.30E-09 | 3.60E-05 | -4.6127 | 3.98E-06 |
| 3 | 47727212 | 49316972 | 292 | 15 | -0.00022368 | 1.39E-09 | 3.73E-05 | -6.0006 | 1.97E-09 |
| 3 | 49316972 | 51832015 | 616 | 26 | -0.00036094 | 2.45E-09 | 4.95E-05 | -7.2852 | 3.21E-13 |
| 4 | 1478711 | 2842979 | 415 | 47 | -0.00015222 | 1.17E-09 | 3.43E-05 | -4.4439 | 8.83E-06 |
| 4 | 100678360 | 103221356 | 1560 | 50 | -0.00029912 | 2.17E-09 | 4.66E-05 | -6.4253 | 1.32E-10 |
| 6 | 97842284 | 100630146 | 1708 | 50 | -0.00033757 | 2.92E-09 | 5.40E-05 | -6.2485 | 4.14E-10 |
| 6 | 108464380 | 110304247 | 988 | 50 | 0.00020015 | 1.91E-09 | 4.37E-05 | 4.577 | 4.72E-06 |
| 11 | 47006137 | 49866050 | 1001 | 34 | -0.00026885 | 2.34E-09 | 4.84E-05 | -5.5563 | 2.76E-08 |
| 15 | 51677560 | 53069096 | 701 | 46 | 0.00020532 | 1.66E-09 | 4.08E-05 | 5.0344 | 4.79E-07 |
| 16 | 27445755 | 29036613 | 461 | 46 | -0.000352 | 2.16E-09 | 4.65E-05 | -7.5674 | 3.81E-14 |
| 17 | 34469036 | 36809344 | 393 | 50 | -0.00019529 | 1.67E-09 | 4.08E-05 | -4.7848 | 1.71E-06 |

**CHR:** chromosome; **SE:** standard error; **SNP:** single-nucleotide polymorphism.

**Supplementary Table 5. Partitioned SNP heritability in BMI**

| **Category** | **Prop. SNPs** | **Prop. h2** | **Prop. h2 SE** | **Enrichment** | **Enrichment SE** | **Enrichment *P*** |
| --- | --- | --- | --- | --- | --- | --- |
| baseL2_0 | 1 | 1 | 6.23E-09 | 1 | 6.23E-09 | NA |
| Coding_UCSCL2_0 | 0.01425914 | 0.082320048 | 0.011775738 | 5.773142612 | 0.825837893 | 6.34E-08 |
| Coding_UCSC.flanking.500L2_0 | 0.049362884 | 0.015160384 | 0.01549801 | 0.307121119 | 0.313960794 | 0.02946593 |
| Conserved_LindbladTohL2_0 | 0.024670538 | 0.205050512 | 0.026707801 | 8.311554106 | 1.082578789 | 1.08E-10 |
| Conserved_LindbladToh.flanking.500L2_0 | 0.305531357 | 0.335108747 | 0.039255482 | 1.096806396 | 0.128482661 | 0.450440839 |
| CTCF_HoffmanL2_0 | 0.023814664 | -0.012036958 | 0.016115591 | -0.505443121 | 0.676708731 | 0.028826733 |
| CTCF_Hoffman.flanking.500L2_0 | 0.04697979 | 0.067912657 | 0.021979311 | 1.445571726 | 0.467846088 | 0.342595267 |
| DGF_ENCODEL2_0 | 0.135362603 | 0.155581701 | 0.052781238 | 1.149369899 | 0.389924812 | 0.69963442 |
| DGF_ENCODE.flanking.500L2_0 | 0.40286092 | 0.411032119 | 0.07499553 | 1.020282928 | 0.18615737 | 0.912919456 |
| DHS_peaks_TrynkaL2_0 | 0.109959825 | 0.14347629 | 0.042637765 | 1.304806457 | 0.387757663 | 0.427821031 |
| DHS_TrynkaL2_0 | 0.165742769 | 0.173750835 | 0.044398525 | 1.048316229 | 0.26787609 | 0.856588861 |
| DHS_Trynka.flanking.500L2_0 | 0.330062661 | 0.43445878 | 0.07678711 | 1.316291819 | 0.232644036 | 0.179561963 |
| Enhancer_AnderssonL2_0 | 0.004313758 | 0.009947383 | 0.006025789 | 2.305966705 | 1.396876878 | 0.352603942 |
| Enhancer_Andersson.flanking.500L2_0 | 0.014708885 | 0.0060436 | 0.009525048 | 0.410880913 | 0.64757104 | 0.366514655 |
| Enhancer_HoffmanL2_0 | 0.041923223 | 0.129002414 | 0.019713236 | 3.077111063 | 0.470222345 | 1.90E-05 |
| Enhancer_Hoffman.flanking.500L2_0 | 0.047882132 | 0.013442039 | 0.023021835 | 0.280731847 | 0.480802221 | 0.136880743 |
| FetalDHS_TrynkaL2_0 | 0.083682888 | 0.114242017 | 0.033466637 | 1.365177752 | 0.399922103 | 0.358206198 |
| FetalDHS_Trynka.flanking.500L2_0 | 0.199517409 | 0.298953461 | 0.043550284 | 1.498382836 | 0.218278116 | 0.025576624 |
| H3K27ac_HniszL2_0 | 0.389043808 | 0.453708605 | 0.019485393 | 1.166214693 | 0.050085345 | 0.000888456 |
| H3K27ac_Hnisz.flanking.500L2_0 | 0.031415367 | 0.052769388 | 0.021752191 | 1.679731693 | 0.692406083 | 0.328449049 |
| H3K27ac_PGC2L2_0 | 0.268521272 | 0.367222601 | 0.029242653 | 1.367573593 | 0.108902558 | 0.000686863 |
| H3K27ac_PGC2.flanking.500L2_0 | 0.066623454 | 0.065817056 | 0.028765427 | 0.987896191 | 0.431761272 | 0.977621333 |
| H3K4me1_peaks_TrynkaL2_0 | 0.168870181 | 0.305683781 | 0.036543271 | 1.810170269 | 0.216398601 | 0.000198387 |
| H3K4me1_TrynkaL2_0 | 0.42345507 | 0.491575433 | 0.041997603 | 1.160867984 | 0.099178416 | 0.099607472 |
| H3K4me1_Trynka.flanking.500L2_0 | 0.1822456 | 0.238423732 | 0.062251073 | 1.308255079 | 0.34157792 | 0.367897694 |
| H3K4me3_peaks_TrynkaL2_0 | 0.041368465 | 0.102066899 | 0.022081525 | 2.467263362 | 0.533776742 | 0.006480864 |
| H3K4me3_TrynkaL2_0 | 0.132886742 | 0.222933861 | 0.02426246 | 1.677623041 | 0.182579987 | 0.000246667 |
| H3K4me3_Trynka.flanking.500L2_0 | 0.122154433 | 0.108190686 | 0.037309405 | 0.885687754 | 0.305428172 | 0.708272441 |
| H3K9ac_peaks_TrynkaL2_0 | 0.038186366 | 0.159944352 | 0.025353295 | 4.188519847 | 0.663935793 | 3.94E-06 |
| H3K9ac_TrynkaL2_0 | 0.125302311 | 0.250544577 | 0.027623488 | 1.999520798 | 0.220454734 | 9.04E-06 |
| H3K9ac_Trynka.flanking.500L2_0 | 0.104469785 | 0.083798636 | 0.031134323 | 0.802132747 | 0.298022277 | 0.507864974 |
| Intron_UCSCL2_0 | 0.387451333 | 0.437142354 | 0.016258249 | 1.128251001 | 0.041962042 | 0.001823289 |
| Intron_UCSC.flanking.500L2_0 | 0.009340298 | 0.047522743 | 0.012889356 | 5.087925869 | 1.379972743 | 0.003784955 |
| PromoterFlanking_HoffmanL2_0 | 0.008276914 | 0.012090408 | 0.011744534 | 1.460738696 | 1.418950787 | 0.744347609 |
| PromoterFlanking_Hoffman.flanking.500L2_0 | 0.024887274 | 0.0338468 | 0.013810159 | 1.360004306 | 0.554908465 | 0.515164794 |
| Promoter_UCSCL2_0 | 0.046335285 | 0.080048326 | 0.020186879 | 1.727588949 | 0.435669689 | 0.093181464 |
| Promoter_UCSC.flanking.500L2_0 | 0.010589887 | 0.005393139 | 0.014713482 | 0.509272611 | 1.389389896 | 0.723294121 |
| Repressed_HoffmanL2_0 | 0.460018094 | 0.446074948 | 0.054265809 | 0.969690005 | 0.117964511 | 0.795662207 |
| Repressed_Hoffman.flanking.500L2_0 | 0.258437495 | 0.189274819 | 0.055078351 | 0.732381419 | 0.213120589 | 0.212842712 |
| SuperEnhancer_HniszL2_0 | 0.167206746 | 0.227820293 | 0.010898831 | 1.362506584 | 0.065181767 | 4.10E-08 |
| SuperEnhancer_Hnisz.flanking.500L2_0 | 0.003173376 | 0.004383256 | 0.005584238 | 1.381259365 | 1.759715166 | 0.828236439 |
| TFBS_ENCODEL2_0 | 0.130971511 | 0.197545611 | 0.03499904 | 1.508309785 | 0.267226361 | 0.055433824 |
| TFBS_ENCODE.flanking.500L2_0 | 0.210069887 | 0.215761405 | 0.044077198 | 1.027093449 | 0.20982159 | 0.897316207 |
| Transcr_HoffmanL2_0 | 0.345192604 | 0.379000565 | 0.041143097 | 1.097939413 | 0.119188813 | 0.408153106 |
| Transcr_Hoffman.flanking.500L2_0 | 0.416710576 | 0.385482938 | 0.062557912 | 0.925061565 | 0.150123168 | 0.613814468 |
| TSS_HoffmanL2_0 | 0.01780694 | 0.042013486 | 0.01201064 | 2.359388343 | 0.674492079 | 0.042408272 |
| TSS_Hoffman.flanking.500L2_0 | 0.016531685 | 0.039918177 | 0.012772447 | 2.414646687 | 0.772604114 | 0.070405767 |
| UTR_3_UCSCL2_0 | 0.01116813 | 0.048803033 | 0.010172442 | 4.369848106 | 0.910845598 | 0.000226344 |
| UTR_3_UCSC.flanking.500L2_0 | 0.015275217 | 0.002459436 | 0.010457824 | 0.161008277 | 0.68462688 | 0.219155431 |
| UTR_5_UCSCL2_0 | 0.00546018 | 0.019553809 | 0.006796222 | 3.581165824 | 1.24468839 | 0.039919303 |
| UTR_5_UCSC.flanking.500L2_0 | 0.021387284 | 0.021806356 | 0.011013583 | 1.019594439 | 0.514959395 | 0.969633342 |
| WeakEnhancer_HoffmanL2_0 | 0.020905666 | 0.047888252 | 0.013798679 | 2.290682877 | 0.660044952 | 0.049333253 |
| WeakEnhancer_Hoffman.flanking.500L2_0 | 0.067818691 | 0.092162842 | 0.022476472 | 1.358959324 | 0.331420017 | 0.281594788 |
| GERP.NSL2_0 | 1.745316256 | 3.095836755 | 0.092318919 | 1.773797009 | 0.052895238 | 7.62E-34 |
| GERP.RSsup4L2_0 | 0.008149925 | 0.086614216 | 0.016779192 | 10.62760867 | 2.058815497 | 6.00E-06 |
| MAFbin1L2_0 | 0.102357276 | 0.047756946 | 0.009003862 | 0.466571089 | 0.087965042 | 8.07E-09 |
| MAFbin2L2_0 | 0.099855414 | 0.051432095 | 0.006607997 | 0.515065666 | 0.066175647 | 6.71E-12 |
| MAFbin3L2_0 | 0.099606637 | 0.055688075 | 0.008309402 | 0.559079969 | 0.08342217 | 5.50E-07 |
| MAFbin4L2_0 | 0.100695687 | 0.064610216 | 0.011108888 | 0.641638362 | 0.110321389 | 0.001718679 |
| MAFbin5L2_0 | 0.098317123 | 0.106503372 | 0.016386956 | 1.083263724 | 0.166674482 | 0.616532213 |
| MAFbin6L2_0 | 0.0996422 | 0.111784444 | 0.01683426 | 1.121858445 | 0.168947095 | 0.471159516 |
| MAFbin7L2_0 | 0.099733793 | 0.158445959 | 0.020151464 | 1.588688782 | 0.202052521 | 0.00443512 |
| MAFbin8L2_0 | 0.100229335 | 0.141838942 | 0.015813529 | 1.415144002 | 0.157773464 | 0.009341328 |
| MAFbin9L2_0 | 0.101099971 | 0.13112717 | 0.01361798 | 1.297005025 | 0.134698159 | 0.027459655 |
| MAFbin10L2_0 | 0.098462564 | 0.130812781 | 0.014537387 | 1.328553468 | 0.147643799 | 0.024340096 |
| MAF_Adj_Predicted_Allele_AgeL2_0 | 3.39E-06 | -0.302488398 | 0.058154111 | -89165.50565 | 17142.27972 | 1.12E-06 |
| MAF_Adj_LLD_AFRL2_0 | 0.002796474 | -0.320069014 | 0.038432744 | -114.4544833 | 13.74328567 | 6.80E-13 |
| Recomb_Rate_10kbL2_0 | 1.552424442 | 1.436637167 | 0.110445897 | 0.925415194 | 0.071144137 | 0.285039794 |
| Nucleotide_Diversity_10kbL2_0 | 4.608695341 | 3.565105959 | 0.125618452 | 0.773560779 | 0.027256836 | 5.76E-13 |
| Backgrd_Selection_StatL2_0 | 0.177719812 | 0.216072771 | 0.005857759 | 1.215805763 | 0.032960644 | 8.90E-11 |
| CpG_Content_50kbL2_0 | 0.010051874 | 0.011396607 | 0.00015866 | 1.133779315 | 0.015784093 | 2.44E-14 |
| MAF_Adj_ASMCL2_0 | -2.35E-14 | -0.455672215 | 0.036360601 | 1.94025E+13 | -1.54823E+12 | 7.51E-24 |
| GTEx_eQTL_MaxCPPL2_0 | 0.010340409 | 0.039905138 | 0.012710377 | 3.85914482 | 1.229194723 | 0.023824862 |
| BLUEPRINT_H3K27acQTL_MaxCPPL2_0 | 0.016545549 | 0.037712754 | 0.00772162 | 2.279329282 | 0.466688652 | 0.005978212 |
| BLUEPRINT_H3K4me1QTL_MaxCPPL2_0 | 0.013372536 | 0.034137734 | 0.007993386 | 2.55282431 | 0.597746457 | 0.00990351 |
| BLUEPRINT_DNA_methylation_MaxCPPL2_0 | 0.031730971 | 0.070670391 | 0.015468113 | 2.22717388 | 0.487476822 | 0.012200332 |
| synonymousL2_0 | 0.003120031 | 0.007591521 | 0.00951757 | 2.433155795 | 3.050473127 | 0.638690511 |
| non_synonymousL2_0 | 0.002715412 | 0.023142797 | 0.006039684 | 8.522758691 | 2.224223997 | 0.001123778 |
| Conserved_Vertebrate_phastCons46wayL2_0 | 0.029436054 | 0.177518518 | 0.024745617 | 6.03064923 | 0.840656732 | 7.90E-09 |
| Conserved_Vertebrate_phastCons46way.flanking.500L2_0 | 0.377534469 | 0.406736516 | 0.039476243 | 1.077349352 | 0.104563281 | 0.459785385 |
| Conserved_Mammal_phastCons46wayL2_0 | 0.021439455 | 0.206045456 | 0.025319668 | 9.610573414 | 1.180984667 | 8.56E-12 |
| Conserved_Mammal_phastCons46way.flanking.500L2_0 | 0.31778015 | 0.32915384 | 0.046739144 | 1.035791066 | 0.147080126 | 0.806815073 |
| Conserved_Primate_phastCons46wayL2_0 | 0.019269072 | 0.219730754 | 0.024412867 | 11.40328699 | 1.266945698 | 1.47E-13 |
| Conserved_Primate_phastCons46way.flanking.500L2_0 | 0.156550261 | 0.253137024 | 0.063289824 | 1.616969664 | 0.404277986 | 0.119501837 |
| BivFlnkL2_0 | 0.013549379 | 0.091478845 | 0.0128805 | 6.751515892 | 0.950634018 | 1.01E-08 |
| BivFlnk.flanking.500L2_0 | 0.017629961 | -0.001230812 | 0.014939611 | -0.06981364 | 0.847399013 | 0.212396343 |
| Human_Promoter_VillarL2_0 | 0.015217007 | 0.045565066 | 0.01055359 | 2.99435131 | 0.693539105 | 0.003923928 |
| Human_Promoter_Villar.flanking.500L2_0 | 0.003588899 | 0.009345298 | 0.008272265 | 2.603945349 | 2.3049588 | 0.485578632 |
| Human_Enhancer_VillarL2_0 | 0.033227431 | 0.034694809 | 0.010064966 | 1.044161634 | 0.302911337 | 0.883999052 |
| Human_Enhancer_Villar.flanking.500L2_0 | 0.009797759 | -0.009952041 | 0.011605902 | -1.015746653 | 1.184546594 | 0.091877956 |
| Ancient_Sequence_Age_Human_PromoterL2_0 | 0.004170665 | 0.026300106 | 0.008644259 | 6.305973462 | 2.072633035 | 0.011388608 |
| Ancient_Sequence_Age_Human_Promoter.flanking.500L2_0 | 0.005320442 | 0.018952763 | 0.009507747 | 3.562253585 | 1.787022132 | 0.148321679 |
| Ancient_Sequence_Age_Human_EnhancerL2_0 | 0.005138095 | 0.019375097 | 0.008273665 | 3.770871847 | 1.610259334 | 0.086432539 |
| Ancient_Sequence_Age_Human_Enhancer.flanking.500L2_0 | 0.009026768 | 0.010859332 | 0.008484549 | 1.203014347 | 0.939932063 | 0.828918117 |
| Human_Enhancer_Villar_Species_Enhancer_CountL2_0 | 0.066283084 | 0.109618074 | 0.03138697 | 1.653786582 | 0.473529116 | 0.169555139 |
| Human_Promoter_Villar_ExACL2_0 | 0.002496662 | 0.024274334 | 0.006257234 | 9.72271499 | 2.506239643 | 0.000585843 |
| Human_Promoter_Villar_ExAC.flanking.500L2_0 | 0.000577069 | 0.003449533 | 0.004535141 | 5.977678169 | 7.858923099 | 0.526473837 |

**SNP:** single-nucleotide polymorphism; **SE:** standard error.

**Supplementary Table 6. Partitioned SNP heritability in cognitive function**

| **Category** | **Prop. SNPs** | **Prop. h2** | **Prop. h2 SE** | **Enrichment** | **Enrichment SE** | **Enrichment *P*** |
| --- | --- | --- | --- | --- | --- | --- |
| baseL2_0 | 1 | 1 | 0 | 1 | 0 | NA |
| Coding_UCSCL2_0 | 0.01425914 | 0.090802223 | 0.013155435 | 6.368001431 | 0.922596645 | 1.59E-08 |
| Coding_UCSC.flanking.500L2_0 | 0.049362884 | -0.001196938 | 0.020234808 | -0.024247732 | 0.409919476 | 0.013638474 |
| Conserved_LindbladTohL2_0 | 0.024670538 | 0.198842928 | 0.028160754 | 8.059934799 | 1.141473054 | 1.89E-09 |
| Conserved_LindbladToh.flanking.500L2_0 | 0.305531357 | 0.368093858 | 0.041413486 | 1.204766219 | 0.135545779 | 0.132138276 |
| CTCF_HoffmanL2_0 | 0.023814664 | -0.010071154 | 0.019700949 | -0.422897184 | 0.827261264 | 0.087442396 |
| CTCF_Hoffman.flanking.500L2_0 | 0.04697979 | 0.07065452 | 0.030016233 | 1.503934337 | 0.638917991 | 0.430384665 |
| DGF_ENCODEL2_0 | 0.135362603 | 0.120283058 | 0.052333773 | 0.88859888 | 0.386619138 | 0.773391534 |
| DGF_ENCODE.flanking.500L2_0 | 0.40286092 | 0.364241279 | 0.064688413 | 0.904136542 | 0.16057257 | 0.550586134 |
| DHS_peaks_TrynkaL2_0 | 0.109959825 | 0.148831763 | 0.050124735 | 1.353510367 | 0.455845897 | 0.439005962 |
| DHS_TrynkaL2_0 | 0.165742769 | 0.195518266 | 0.058595189 | 1.179648844 | 0.353530895 | 0.611516719 |
| DHS_Trynka.flanking.500L2_0 | 0.330062661 | 0.376713135 | 0.062943749 | 1.141338237 | 0.190702423 | 0.459360465 |
| Enhancer_AnderssonL2_0 | 0.004313758 | -0.001902019 | 0.008550535 | -0.440919275 | 1.982154419 | 0.467923047 |
| Enhancer_Andersson.flanking.500L2_0 | 0.014708885 | 0.015481429 | 0.012181518 | 1.052522325 | 0.828174153 | 0.94947563 |
| Enhancer_HoffmanL2_0 | 0.041923223 | 0.079508655 | 0.022013461 | 1.896530114 | 0.525089887 | 0.086852426 |
| Enhancer_Hoffman.flanking.500L2_0 | 0.047882132 | 0.025993658 | 0.025953348 | 0.542867609 | 0.542025743 | 0.400228458 |
| FetalDHS_TrynkaL2_0 | 0.083682888 | 0.200732588 | 0.041490052 | 2.398729219 | 0.495800907 | 0.005373105 |
| FetalDHS_Trynka.flanking.500L2_0 | 0.199517409 | 0.219403111 | 0.048150703 | 1.099669007 | 0.241335848 | 0.679951418 |
| H3K27ac_HniszL2_0 | 0.389043808 | 0.456713284 | 0.020027115 | 1.173937932 | 0.051477788 | 0.000903771 |
| H3K27ac_Hnisz.flanking.500L2_0 | 0.031415367 | 0.039438964 | 0.023800656 | 1.255403555 | 0.757611892 | 0.736234146 |
| H3K27ac_PGC2L2_0 | 0.268521272 | 0.343115152 | 0.030433626 | 1.277795047 | 0.113337857 | 0.014266874 |
| H3K27ac_PGC2.flanking.500L2_0 | 0.066623454 | 0.089279884 | 0.033843177 | 1.340066888 | 0.507976923 | 0.504309598 |
| H3K4me1_peaks_TrynkaL2_0 | 0.168870181 | 0.324652233 | 0.048334816 | 1.922495912 | 0.286224692 | 0.001368068 |
| H3K4me1_TrynkaL2_0 | 0.42345507 | 0.55830014 | 0.043279684 | 1.318440089 | 0.102206084 | 0.002200741 |
| H3K4me1_Trynka.flanking.500L2_0 | 0.1822456 | 0.146282903 | 0.04906026 | 0.80266905 | 0.269198597 | 0.463657236 |
| H3K4me3_peaks_TrynkaL2_0 | 0.041368465 | 0.087474881 | 0.031916646 | 2.114530483 | 0.771521142 | 0.149219059 |
| H3K4me3_TrynkaL2_0 | 0.132886742 | 0.225518685 | 0.029255663 | 1.697074372 | 0.220154868 | 0.001856549 |
| H3K4me3_Trynka.flanking.500L2_0 | 0.122154433 | 0.085779716 | 0.037683562 | 0.702223518 | 0.308491152 | 0.338110332 |
| H3K9ac_peaks_TrynkaL2_0 | 0.038186366 | 0.130051896 | 0.027739388 | 3.405715416 | 0.726421249 | 0.001074259 |
| H3K9ac_TrynkaL2_0 | 0.125302311 | 0.237367505 | 0.02483418 | 1.894358551 | 0.19819411 | 8.59E-06 |
| H3K9ac_Trynka.flanking.500L2_0 | 0.104469785 | 0.061545335 | 0.036725412 | 0.589120911 | 0.351540995 | 0.243786035 |
| Intron_UCSCL2_0 | 0.387451333 | 0.425360167 | 0.016511822 | 1.097841538 | 0.042616507 | 0.023956078 |
| Intron_UCSC.flanking.500L2_0 | 0.009340298 | 0.064834441 | 0.01411249 | 6.941367731 | 1.5109251 | 0.000104238 |
| PromoterFlanking_HoffmanL2_0 | 0.008276914 | 0.022722334 | 0.013233225 | 2.745266459 | 1.598811433 | 0.276314305 |
| PromoterFlanking_Hoffman.flanking.500L2_0 | 0.024887274 | 0.015158752 | 0.017926582 | 0.609096508 | 0.720311198 | 0.587953798 |
| Promoter_UCSCL2_0 | 0.046335285 | 0.100563759 | 0.017524304 | 2.170349424 | 0.378206469 | 0.001861602 |
| Promoter_UCSC.flanking.500L2_0 | 0.010589887 | -0.001030778 | 0.01492386 | -0.097336053 | 1.409255792 | 0.436342535 |
| Repressed_HoffmanL2_0 | 0.460018094 | 0.37462543 | 0.05002036 | 0.814371076 | 0.108735636 | 0.089544381 |
| Repressed_Hoffman.flanking.500L2_0 | 0.258437495 | 0.274403933 | 0.055428952 | 1.061780657 | 0.214477205 | 0.7734171 |
| SuperEnhancer_HniszL2_0 | 0.167206746 | 0.209575225 | 0.011072853 | 1.253389766 | 0.066222524 | 0.000162412 |
| SuperEnhancer_Hnisz.flanking.500L2_0 | 0.003173376 | 0.007767531 | 0.007208462 | 2.447718364 | 2.271543603 | 0.524562817 |
| TFBS_ENCODEL2_0 | 0.130971511 | 0.147402084 | 0.041510311 | 1.125451507 | 0.316941529 | 0.692232217 |
| TFBS_ENCODE.flanking.500L2_0 | 0.210069887 | 0.207096361 | 0.055190794 | 0.985845062 | 0.262725869 | 0.957051618 |
| Transcr_HoffmanL2_0 | 0.345192604 | 0.457625184 | 0.040693845 | 1.325709701 | 0.11788736 | 0.006408331 |
| Transcr_Hoffman.flanking.500L2_0 | 0.416710576 | 0.274354006 | 0.051828 | 0.658380232 | 0.124374094 | 0.006496311 |
| TSS_HoffmanL2_0 | 0.01780694 | 0.051287164 | 0.01518417 | 2.880178436 | 0.852710797 | 0.029323455 |
| TSS_Hoffman.flanking.500L2_0 | 0.016531685 | 0.020016667 | 0.017079046 | 1.210806263 | 1.033109866 | 0.838310497 |
| UTR_3_UCSCL2_0 | 0.01116813 | 0.041989523 | 0.010589081 | 3.75976304 | 0.948151684 | 0.003573732 |
| UTR_3_UCSC.flanking.500L2_0 | 0.015275217 | 0.002397149 | 0.009442824 | 0.156930589 | 0.618179363 | 0.174184316 |
| UTR_5_UCSCL2_0 | 0.00546018 | 0.022722899 | 0.007984757 | 4.161566033 | 1.462361582 | 0.031214726 |
| UTR_5_UCSC.flanking.500L2_0 | 0.021387284 | 0.01971414 | 0.012751343 | 0.921769213 | 0.596211426 | 0.895745591 |
| WeakEnhancer_HoffmanL2_0 | 0.020905666 | 0.066570448 | 0.019967227 | 3.184325603 | 0.955110781 | 0.023214675 |
| WeakEnhancer_Hoffman.flanking.500L2_0 | 0.067818691 | 0.091971062 | 0.028742079 | 1.356131482 | 0.423807638 | 0.400431611 |
| GERP.NSL2_0 | 1.745316256 | 3.401997057 | 0.11555366 | 1.949215247 | 0.066207863 | 4.01E-35 |
| GERP.RSsup4L2_0 | 0.008149925 | 0.08148291 | 0.019470631 | 9.997994836 | 2.389056437 | 0.000188492 |
| MAFbin1L2_0 | 0.102357276 | 0.069969082 | 0.009463766 | 0.683577019 | 0.092458165 | 0.000565943 |
| MAFbin2L2_0 | 0.099855414 | 0.031609847 | 0.009884327 | 0.316556162 | 0.098986392 | 3.78E-11 |
| MAFbin3L2_0 | 0.099606637 | 0.070819143 | 0.011350699 | 0.710988192 | 0.113955245 | 0.011152157 |
| MAFbin4L2_0 | 0.100695687 | 0.063489606 | 0.011334209 | 0.63050969 | 0.112559036 | 0.001597751 |
| MAFbin5L2_0 | 0.098317123 | 0.103528124 | 0.013016008 | 1.053001975 | 0.132388012 | 0.689363726 |
| MAFbin6L2_0 | 0.0996422 | 0.097806745 | 0.015838202 | 0.981579533 | 0.158950745 | 0.907752238 |
| MAFbin7L2_0 | 0.099733793 | 0.114082079 | 0.015857907 | 1.143865838 | 0.159002347 | 0.367092423 |
| MAFbin8L2_0 | 0.100229335 | 0.13666567 | 0.016180334 | 1.363529654 | 0.161433114 | 0.024582054 |
| MAFbin9L2_0 | 0.101099971 | 0.147291184 | 0.016161758 | 1.456886517 | 0.159859173 | 0.004578302 |
| MAFbin10L2_0 | 0.098462564 | 0.16473852 | 0.016703813 | 1.67310817 | 0.169646329 | 8.62E-05 |
| MAF_Adj_Predicted_Allele_AgeL2_0 | 3.39E-06 | -0.34666756 | 0.054992477 | -102188.3433 | 16210.3145 | 1.14E-08 |
| MAF_Adj_LLD_AFRL2_0 | 0.002796474 | -0.315174955 | 0.03309789 | -112.7044012 | 11.83557853 | 1.40E-15 |
| Recomb_Rate_10kbL2_0 | 1.552424442 | 1.314426683 | 0.094163214 | 0.846692855 | 0.060655586 | 0.009805709 |
| Nucleotide_Diversity_10kbL2_0 | 4.608695341 | 3.515937809 | 0.083478915 | 0.762892218 | 0.018113351 | 3.81E-28 |
| Backgrd_Selection_StatL2_0 | 0.177719812 | 0.222372944 | 0.005623285 | 1.251255792 | 0.031641294 | 2.00E-14 |
| CpG_Content_50kbL2_0 | 0.010051874 | 0.010943663 | 0.000131173 | 1.088718713 | 0.013049582 | 2.57E-10 |
| MAF_Adj_ASMCL2_0 | -2.35E-14 | -0.526143245 | 0.043575819 | 2.24E+13 | -1.86E+12 | 8.55E-25 |
| GTEx_eQTL_MaxCPPL2_0 | 0.010340409 | 0.045615537 | 0.008688387 | 4.411385946 | 0.840236287 | 6.93E-05 |
| BLUEPRINT_H3K27acQTL_MaxCPPL2_0 | 0.016545549 | 0.031057634 | 0.009126487 | 1.877099014 | 0.551597703 | 0.112424236 |
| BLUEPRINT_H3K4me1QTL_MaxCPPL2_0 | 0.013372536 | 0.021889276 | 0.008233933 | 1.636882987 | 0.615734625 | 0.304808685 |
| BLUEPRINT_DNA_methylation_MaxCPPL2_0 | 0.031730971 | 0.031283551 | 0.01112165 | 0.985899552 | 0.350498249 | 0.967889859 |
| synonymousL2_0 | 0.003120031 | 0.034572299 | 0.010085901 | 11.08075557 | 3.232628759 | 0.001980633 |
| non_synonymousL2_0 | 0.002715412 | 0.009960809 | 0.006552686 | 3.668250225 | 2.413146636 | 0.271033323 |
| Conserved_Vertebrate_phastCons46wayL2_0 | 0.029436054 | 0.194686674 | 0.027503498 | 6.613884852 | 0.934347302 | 6.79E-09 |
| Conserved_Vertebrate_phastCons46way.flanking.500L2_0 | 0.377534469 | 0.358856286 | 0.050015868 | 0.950525886 | 0.132480269 | 0.709529103 |
| Conserved_Mammal_phastCons46wayL2_0 | 0.021439455 | 0.18951515 | 0.026879624 | 8.839550735 | 1.253745696 | 2.15E-09 |
| Conserved_Mammal_phastCons46way.flanking.500L2_0 | 0.31778015 | 0.326219914 | 0.047187722 | 1.026558501 | 0.148491723 | 0.858047125 |
| Conserved_Primate_phastCons46wayL2_0 | 0.019269072 | 0.246518535 | 0.031503638 | 12.7934827 | 1.634932816 | 4.04E-12 |
| Conserved_Primate_phastCons46way.flanking.500L2_0 | 0.156550261 | 0.265842633 | 0.040328194 | 1.698129599 | 0.257605411 | 0.007207109 |
| BivFlnkL2_0 | 0.013549379 | 0.04687759 | 0.013381494 | 3.459759404 | 0.987609425 | 0.013438397 |
| BivFlnk.flanking.500L2_0 | 0.017629961 | 0.02521986 | 0.016110711 | 1.430511393 | 0.913825706 | 0.637765643 |
| Human_Promoter_VillarL2_0 | 0.015217007 | 0.045184713 | 0.010989048 | 2.969356073 | 0.722155689 | 0.007001617 |
| Human_Promoter_Villar.flanking.500L2_0 | 0.003588899 | 0.019012981 | 0.009457808 | 5.297719042 | 2.635294849 | 0.101585613 |
| Human_Enhancer_VillarL2_0 | 0.033227431 | 0.024615622 | 0.011100752 | 0.740822297 | 0.334083968 | 0.437978859 |
| Human_Enhancer_Villar.flanking.500L2_0 | 0.009797759 | 0.006839914 | 0.011001009 | 0.698109992 | 1.12280864 | 0.78839032 |
| Ancient_Sequence_Age_Human_PromoterL2_0 | 0.004170665 | 0.032544462 | 0.010415072 | 7.803182133 | 2.497220672 | 0.007221405 |
| Ancient_Sequence_Age_Human_Promoter.flanking.500L2_0 | 0.005320442 | 0.032842621 | 0.010794753 | 6.172912198 | 2.028920396 | 0.010761094 |
| Ancient_Sequence_Age_Human_EnhancerL2_0 | 0.005138095 | 0.021903516 | 0.009987502 | 4.262964575 | 1.943814215 | 0.093845884 |
| Ancient_Sequence_Age_Human_Enhancer.flanking.500L2_0 | 0.009026768 | -0.006035234 | 0.011359251 | -0.668592957 | 1.258396179 | 0.184157334 |
| Human_Enhancer_Villar_Species_Enhancer_CountL2_0 | 0.066283084 | 0.125980132 | 0.030458181 | 1.900637763 | 0.459516659 | 0.051124309 |
| Human_Promoter_Villar_ExACL2_0 | 0.002496662 | 0.016458548 | 0.005735792 | 6.59222059 | 2.297383974 | 0.015444483 |
| Human_Promoter_Villar_ExAC.flanking.500L2_0 | 0.000577069 | 0.004369962 | 0.004176595 | 7.572685223 | 7.237600735 | 0.363504539 |

**SNP:** single-nucleotide polymorphism; **SE:** standard error.

**Supplementary Table 7. Partitioned SNP heritability in BMI and cognitive function**

| **Category** | **Prop. SNPs** | **Prop. h2** | **Prop. h2 SE** | **Enrichment** | **Enrichment SE** | **Enrichment *P*** |
| --- | --- | --- | --- | --- | --- | --- |
| baseL2_0 | 1 | 1 | 0 | 1 | 0 | NA |
| Coding_UCSCL2_0 | 0.01425914 | 0.090802223 | 0.013155435 | 6.368001431 | 0.922596645 | 1.59E-08 |
| Coding_UCSC.flanking.500L2_0 | 0.049362884 | -0.001196938 | 0.020234808 | -0.024247732 | 0.409919476 | 0.013638474 |
| Conserved_LindbladTohL2_0 | 0.024670538 | 0.198842928 | 0.028160754 | 8.059934799 | 1.141473054 | 1.89E-09 |
| H3K27ac_HniszL2_0 | 0.389043808 | 0.456713284 | 0.020027115 | 1.173937932 | 0.051477788 | 0.000903771 |
| H3K27ac_PGC2L2_0 | 0.268521272 | 0.343115152 | 0.030433626 | 1.277795047 | 0.113337857 | 0.014266874 |
| H3K4me1_peaks_TrynkaL2_0 | 0.168870181 | 0.324652233 | 0.048334816 | 1.922495912 | 0.286224692 | 0.001368068 |
| H3K4me1_TrynkaL2_0 | 0.42345507 | 0.55830014 | 0.043279684 | 1.318440089 | 0.102206084 | 0.002200741 |
| Intron_UCSCL2_0 | 0.387451333 | 0.425360167 | 0.016511822 | 1.097841538 | 0.042616507 | 0.023956078 |
| Intron_UCSC.flanking.500L2_0 | 0.009340298 | 0.064834441 | 0.01411249 | 6.941367731 | 1.5109251 | 0.000104238 |
| SuperEnhancer_HniszL2_0 | 0.167206746 | 0.209575225 | 0.011072853 | 1.253389766 | 0.066222524 | 0.000162412 |
| TSS_HoffmanL2_0 | 0.01780694 | 0.051287164 | 0.01518417 | 2.880178436 | 0.852710797 | 0.029323455 |
| TSS_Hoffman.flanking.500L2_0 | 0.016531685 | 0.020016667 | 0.017079046 | 1.210806263 | 1.033109866 | 0.838310497 |
| UTR_3_UCSCL2_0 | 0.01116813 | 0.041989523 | 0.010589081 | 3.75976304 | 0.948151684 | 0.003573732 |
| UTR_5_UCSCL2_0 | 0.00546018 | 0.022722899 | 0.007984757 | 4.161566033 | 1.462361582 | 0.031214726 |
| WeakEnhancer_HoffmanL2_0 | 0.020905666 | 0.066570448 | 0.019967227 | 3.184325603 | 0.955110781 | 0.023214675 |
| GERP.NSL2_0 | 1.745316256 | 3.401997057 | 0.11555366 | 1.949215247 | 0.066207863 | 4.01E-35 |
| GERP.RSsup4L2_0 | 0.008149925 | 0.08148291 | 0.019470631 | 9.997994836 | 2.389056437 | 0.000188492 |
| MAFbin1L2_0 | 0.102357276 | 0.069969082 | 0.009463766 | 0.683577019 | 0.092458165 | 0.000565943 |
| MAFbin2L2_0 | 0.099855414 | 0.031609847 | 0.009884327 | 0.316556162 | 0.098986392 | 3.78E-11 |
| MAFbin3L2_0 | 0.099606637 | 0.070819143 | 0.011350699 | 0.710988192 | 0.113955245 | 0.011152157 |
| MAFbin4L2_0 | 0.100695687 | 0.063489606 | 0.011334209 | 0.63050969 | 0.112559036 | 0.001597751 |
| MAFbin8L2_0 | 0.100229335 | 0.13666567 | 0.016180334 | 1.363529654 | 0.161433114 | 0.024582054 |
| MAFbin9L2_0 | 0.101099971 | 0.147291184 | 0.016161758 | 1.456886517 | 0.159859173 | 0.004578302 |
| MAFbin10L2_0 | 0.098462564 | 0.16473852 | 0.016703813 | 1.67310817 | 0.169646329 | 8.62E-05 |
| MAF_Adj_Predicted_Allele_AgeL2_0 | 3.39E-06 | -0.34666756 | 0.054992477 | -102188.3433 | 16210.3145 | 1.14E-08 |
| MAF_Adj_LLD_AFRL2_0 | 0.002796474 | -0.315174955 | 0.03309789 | -112.7044012 | 11.83557853 | 1.40E-15 |
| Nucleotide_Diversity_10kbL2_0 | 4.608695341 | 3.515937809 | 0.083478915 | 0.762892218 | 0.018113351 | 3.81E-28 |
| Backgrd_Selection_StatL2_0 | 0.177719812 | 0.222372944 | 0.005623285 | 1.251255792 | 0.031641294 | 2.00E-14 |
| CpG_Content_50kbL2_0 | 0.010051874 | 0.010943663 | 0.000131173 | 1.088718713 | 0.013049582 | 2.57E-10 |
| MAF_Adj_ASMCL2_0 | -2.35E-14 | -0.526143245 | 0.043575819 | 2.24E+13 | -1.86E+12 | 8.55E-25 |
| GTEx_eQTL_MaxCPPL2_0 | 0.010340409 | 0.045615537 | 0.008688387 | 4.411385946 | 0.840236287 | 6.93E-05 |
| synonymousL2_0 | 0.003120031 | 0.034572299 | 0.010085901 | 11.08075557 | 3.232628759 | 0.001980633 |
| Conserved_Vertebrate_phastCons46wayL2_0 | 0.029436054 | 0.194686674 | 0.027503498 | 6.613884852 | 0.934347302 | 6.79E-09 |
| Conserved_Mammal_phastCons46wayL2_0 | 0.021439455 | 0.18951515 | 0.026879624 | 8.839550735 | 1.253745696 | 2.15E-09 |
| Conserved_Primate_phastCons46wayL2_0 | 0.019269072 | 0.246518535 | 0.031503638 | 12.7934827 | 1.634932816 | 4.04E-12 |
| BivFlnkL2_0 | 0.013549379 | 0.04687759 | 0.013381494 | 3.459759404 | 0.987609425 | 0.013438397 |
| Human_Promoter_VillarL2_0 | 0.015217007 | 0.045184713 | 0.010989048 | 2.969356073 | 0.722155689 | 0.007001617 |
| Ancient_Sequence_Age_Human_PromoterL2_0 | 0.004170665 | 0.032544462 | 0.010415072 | 7.803182133 | 2.497220672 | 0.007221405 |
| Human_Promoter_Villar_ExACL2_0 | 0.002496662 | 0.016458548 | 0.005735792 | 6.59222059 | 2.297383974 | 0.015444483 |

**SNP:** single-nucleotide polymorphism; **SE:** standard error.

**Supplementary Table 8.** **Summary of Mendelian randomization results between body mass index and cognitive function**

| **BMI to cognitive function** | | | | | | |
| --- | --- | --- | --- | --- | --- | --- |
| **Method** | **SNPs** | **β** | **SE** | ***P*** | **Q** | **Q_*P*** |
| MR Egger | 172 | -0.19 | 0.19 | 0.298 | 604.4366 | 9.279071e-50 |
| Weighted median | 172 | -0.15 | 0.04 | <0.05 | 604.5965 | 5.193872e-50 |
| Inverse variance weighted | 172 | -0.16 | 0.04 | <0.05 |  |  |
| Simple mode | 172 | -0.28 | 0.14 | <0.05 |  |  |
| Weighted mode | 172 | -0.27 | 0.14 | 0.058 |  |  |
| GSMR | 1184 (368) | -0.05 | 0.01 | <0.05 |  |  |
| **cognitive function to BMI** | | | | | | |
| **Method** | **SNPs** | **β** | **SE** | ***P*** | **Q** | **Q_*P*** |
| MR Egger | 41 | 0.03 | 0.17 | 0.871 | 228.6262 | 2.826381e-28 |
| Weighted median | 41 | -0.01 | 0.03 | 0.666 | 228.1340 | 1.418823e-28 |
| Inverse variance weighted | 41 | -0.02 | 0.04 | 0.565 |  |  |
| Simple mode | 41 | -0.07 | 0.07 | 0.371 |  |  |
| Weighted mode | 41 | -0.05 | 0.08 | 0.559 |  |  |
| GSMR | 136  (23) | -0.14 | 0.01 | <0.05 |  |  |

**BMI:** body mass index; **SNP:** single-nucleotide polymorphism; **SE:** standard error. Numbers in the brackets of GSMR results indicate the numbers of SNPs remained after the HEIDI (Heterogeneity In Dependent Instrument) test.

**Supplementary Table 9. Heritability enrichment of BMI and cognitive function in specific tissues**

| **Name** | **Trait** | **Coefficient** | **Coefficient SE** | **Coefficient *P*** |
| --- | --- | --- | --- | --- |
| Brain_Frontal_Cortex_(BA9) | BMI | 7.16E-09 | 2.40E-09 | 0.001406492 |
| Brain_Putamen_(basal_ganglia) | BMI | 6.60E-09 | 2.41E-09 | 0.003135937 |
| Brain_Nucleus_accumbens_(basal_ganglia) | BMI | 6.49E-09 | 2.47E-09 | 0.0043315 |
| Brain_Anterior_cingulate_cortex_(BA24) | BMI | 6.18E-09 | 2.44E-09 | 0.005661728 |
| Brain_Caudate_(basal_ganglia) | BMI | 6.12E-09 | 2.48E-09 | 0.006776172 |
| Brain_Hypothalamus | BMI | 5.42E-09 | 2.40E-09 | 0.012023621 |
| Brain_Cerebellar_Hemisphere | BMI | 4.47E-09 | 2.02E-09 | 0.013404829 |
| Brain_Hippocampus | BMI | 3.82E-09 | 2.01E-09 | 0.028674823 |
| Brain_Cerebellum | BMI | 3.79E-09 | 2.05E-09 | 0.032195008 |
| Brain_Amygdala | BMI | 3.13E-09 | 1.98E-09 | 0.056874851 |
| Brain_Cortex | BMI | 3.35E-09 | 2.18E-09 | 0.062154723 |
| Brain_Substantia_nigra | BMI | 2.72E-09 | 2.04E-09 | 0.090590874 |
| Pancreas | BMI | 2.69E-09 | 2.28E-09 | 0.119700528 |
| Pituitary | BMI | 2.27E-09 | 2.65E-09 | 0.196024026 |
| Artery_Tibial | BMI | 1.15E-09 | 2.45E-09 | 0.320210289 |
| Testis | BMI | 1.81E-09 | 4.63E-09 | 0.347924641 |
| Liver | BMI | 7.14E-10 | 2.48E-09 | 0.386897687 |
| Brain_Spinal_cord_(cervical_c-1) | BMI | 3.03E-10 | 1.91E-09 | 0.43698116 |
| Small_Intestine_Terminal_Ileum | BMI | -1.30E-09 | 2.72E-09 | 0.684142225 |
| Esophagus_Muscularis | BMI | -1.16E-09 | 2.26E-09 | 0.695752347 |
| Nerve_Tibial | BMI | -1.39E-09 | 2.60E-09 | 0.703502122 |
| Spleen | BMI | -1.72E-09 | 3.11E-09 | 0.710306564 |
| Heart_Left_Ventricle | BMI | -1.42E-09 | 2.29E-09 | 0.732848102 |
| Ovary | BMI | -1.67E-09 | 2.68E-09 | 0.732991393 |
| Adrenal_Gland | BMI | -1.88E-09 | 3.01E-09 | 0.734149722 |
| Esophagus_Gastroesophageal_Junction | BMI | -1.83E-09 | 2.30E-09 | 0.786531768 |
| Whole_Blood | BMI | -2.14E-09 | 2.48E-09 | 0.80570238 |
| Kidney_Cortex | BMI | -2.82E-09 | 2.66E-09 | 0.856109238 |
| Cervix_Endocervix | BMI | -3.39E-09 | 2.95E-09 | 0.874717224 |
| Stomach | BMI | -2.70E-09 | 2.33E-09 | 0.876734706 |
| Uterus | BMI | -3.01E-09 | 2.59E-09 | 0.877894882 |
| Fallopian_Tube | BMI | -3.21E-09 | 2.72E-09 | 0.881385161 |
| Adipose_Subcutaneous | BMI | -3.20E-09 | 2.18E-09 | 0.928831124 |
| Prostate | BMI | -3.48E-09 | 2.35E-09 | 0.930585055 |
| Thyroid | BMI | -3.41E-09 | 2.13E-09 | 0.945538557 |
| Skin_Sun_Exposed_(Lower_leg) | BMI | -3.77E-09 | 2.31E-09 | 0.948462447 |
| Colon_Transverse | BMI | -3.88E-09 | 2.26E-09 | 0.956725097 |
| Breast_Mammary_Tissue | BMI | -4.18E-09 | 2.35E-09 | 0.962323451 |
| Bladder | BMI | -3.54E-09 | 1.98E-09 | 0.963056026 |
| Cells_EBV-transformed_lymphocytes | BMI | -4.52E-09 | 2.33E-09 | 0.973941972 |
| Muscle_Skeletal | BMI | -3.64E-09 | 1.85E-09 | 0.975216194 |
| Skin_Not_Sun_Exposed_(Suprapubic) | BMI | -4.34E-09 | 2.11E-09 | 0.980091469 |
| Esophagus_Mucosa | BMI | -4.66E-09 | 2.22E-09 | 0.98236428 |
| Artery_Coronary | BMI | -4.16E-09 | 1.69E-09 | 0.993158903 |
| Artery_Aorta | BMI | -5.04E-09 | 1.92E-09 | 0.995704956 |
| Colon_Sigmoid | BMI | -5.39E-09 | 2.02E-09 | 0.996191368 |
| Cells_Transformed_fibroblasts | BMI | -6.40E-09 | 2.38E-09 | 0.996370161 |
| Minor_Salivary_Gland | BMI | -6.07E-09 | 2.16E-09 | 0.997567174 |
| Heart_Atrial_Appendage | BMI | -6.30E-09 | 1.93E-09 | 0.999444496 |
| Adipose_Visceral_(Omentum) | BMI | -7.14E-09 | 2.19E-09 | 0.999453345 |
| Cervix_Ectocervix | BMI | -7.96E-09 | 2.13E-09 | 0.999907585 |
| Lung | BMI | -7.74E-09 | 1.87E-09 | 0.999981974 |
| Vagina | BMI | -9.10E-09 | 2.18E-09 | 0.999984853 |
| Brain_Frontal_Cortex_(BA9) | cognitive function | 1.19E-08 | 2.24E-09 | 4.83E-08 |
| Brain_Cortex | cognitive function | 1.12E-08 | 2.28E-09 | 4.37E-07 |
| Brain_Anterior_cingulate_cortex_(BA24) | cognitive function | 1.04E-08 | 2.24E-09 | 1.75E-06 |
| Brain_Hippocampus | cognitive function | 8.81E-09 | 2.32E-09 | 7.34E-05 |
| Brain_Amygdala | cognitive function | 8.59E-09 | 2.28E-09 | 8.11E-05 |
| Brain_Nucleus_accumbens_(basal_ganglia) | cognitive function | 8.88E-09 | 2.35E-09 | 8.13E-05 |
| Brain_Hypothalamus | cognitive function | 7.76E-09 | 2.28E-09 | 0.000326364 |
| Brain_Substantia_nigra | cognitive function | 7.15E-09 | 2.25E-09 | 0.000739263 |
| Brain_Putamen_(basal_ganglia) | cognitive function | 7.14E-09 | 2.29E-09 | 0.00090543 |
| Brain_Caudate_(basal_ganglia) | cognitive function | 7.16E-09 | 2.30E-09 | 0.000942305 |
| Brain_Cerebellum | cognitive function | 5.34E-09 | 1.99E-09 | 0.003608106 |
| Brain_Cerebellar_Hemisphere | cognitive function | 5.38E-09 | 2.04E-09 | 0.004222436 |
| Brain_Spinal_cord_(cervical_c-1) | cognitive function | 5.13E-09 | 2.21E-09 | 0.010154429 |
| Muscle_Skeletal | cognitive function | 1.70E-09 | 2.50E-09 | 0.24856482 |
| Whole_Blood | cognitive function | 1.18E-09 | 2.18E-09 | 0.293154336 |
| Uterus | cognitive function | 5.72E-10 | 2.43E-09 | 0.406926342 |
| Cells_EBV-transformed_lymphocytes | cognitive function | -6.37E-10 | 2.43E-09 | 0.603543995 |
| Pituitary | cognitive function | -9.07E-10 | 2.25E-09 | 0.656615433 |
| Small_Intestine_Terminal_Ileum | cognitive function | -1.19E-09 | 2.31E-09 | 0.696967776 |
| Nerve_Tibial | cognitive function | -1.11E-09 | 2.11E-09 | 0.701031099 |
| Artery_Tibial | cognitive function | -1.22E-09 | 2.23E-09 | 0.707942066 |
| Cervix_Endocervix | cognitive function | -1.32E-09 | 2.15E-09 | 0.730622954 |
| Artery_Coronary | cognitive function | -1.51E-09 | 2.29E-09 | 0.744544018 |
| Prostate | cognitive function | -1.91E-09 | 2.69E-09 | 0.761055505 |
| Artery_Aorta | cognitive function | -1.74E-09 | 2.28E-09 | 0.776466025 |
| Fallopian_Tube | cognitive function | -2.10E-09 | 2.33E-09 | 0.81642154 |
| Esophagus_Muscularis | cognitive function | -2.49E-09 | 2.36E-09 | 0.854075227 |
| Bladder | cognitive function | -2.41E-09 | 2.15E-09 | 0.869625074 |
| Spleen | cognitive function | -2.79E-09 | 2.33E-09 | 0.884327233 |
| Skin_Not_Sun_Exposed_(Suprapubic) | cognitive function | -3.13E-09 | 2.50E-09 | 0.894096692 |
| Lung | cognitive function | -3.00E-09 | 2.24E-09 | 0.909532394 |
| Ovary | cognitive function | -3.10E-09 | 2.30E-09 | 0.911181277 |
| Colon_Transverse | cognitive function | -3.08E-09 | 2.25E-09 | 0.914638532 |
| Adipose_Subcutaneous | cognitive function | -3.51E-09 | 2.24E-09 | 0.941166581 |
| Kidney_Cortex | cognitive function | -3.47E-09 | 2.20E-09 | 0.942602983 |
| Skin_Sun_Exposed_(Lower_leg) | cognitive function | -3.74E-09 | 2.34E-09 | 0.944776476 |
| Pancreas | cognitive function | -3.22E-09 | 1.94E-09 | 0.95181826 |
| Thyroid | cognitive function | -3.57E-09 | 2.13E-09 | 0.953479643 |
| Breast_Mammary_Tissue | cognitive function | -3.61E-09 | 2.09E-09 | 0.957965349 |
| Esophagus_Gastroesophageal_Junction | cognitive function | -4.10E-09 | 2.37E-09 | 0.95798492 |
| Heart_Left_Ventricle | cognitive function | -3.39E-09 | 1.90E-09 | 0.96284047 |
| Colon_Sigmoid | cognitive function | -4.57E-09 | 2.50E-09 | 0.966267606 |
| Testis | cognitive function | -3.64E-09 | 1.91E-09 | 0.971531984 |
| Minor_Salivary_Gland | cognitive function | -4.98E-09 | 2.41E-09 | 0.9807922 |
| Adipose_Visceral_(Omentum) | cognitive function | -5.28E-09 | 2.40E-09 | 0.986267824 |
| Esophagus_Mucosa | cognitive function | -4.92E-09 | 2.08E-09 | 0.99105072 |
| Cervix_Ectocervix | cognitive function | -4.88E-09 | 2.06E-09 | 0.991111394 |
| Heart_Atrial_Appendage | cognitive function | -4.62E-09 | 1.93E-09 | 0.991547439 |
| Cells_Transformed_fibroblasts | cognitive function | -6.04E-09 | 2.25E-09 | 0.996438584 |
| Liver | cognitive function | -5.94E-09 | 2.20E-09 | 0.996489605 |
| Stomach | cognitive function | -6.13E-09 | 2.06E-09 | 0.998557988 |
| Adrenal_Gland | cognitive function | -6.88E-09 | 2.21E-09 | 0.99906398 |
| Vagina | cognitive function | -6.50E-09 | 1.84E-09 | 0.999802258 |

**SE:** standard error.
