# Supplementary material for: Investigating causality and shared genetic architecture between body mass index and cognitive function: a genome-wide cross-trait analysis and bi-directional Mendelian randomization study
Source: Front Aging Neurosci. 2024 Oct 16;16:1466799. doi: 10.3389/fnagi.2024.1466799 (PMC11522962; doi:10.3389/fnagi.2024.1466799)
Supplement: Supplementary file 2 [file Presentation_1.pdf]

Supplementary Material

Supplementary Figures

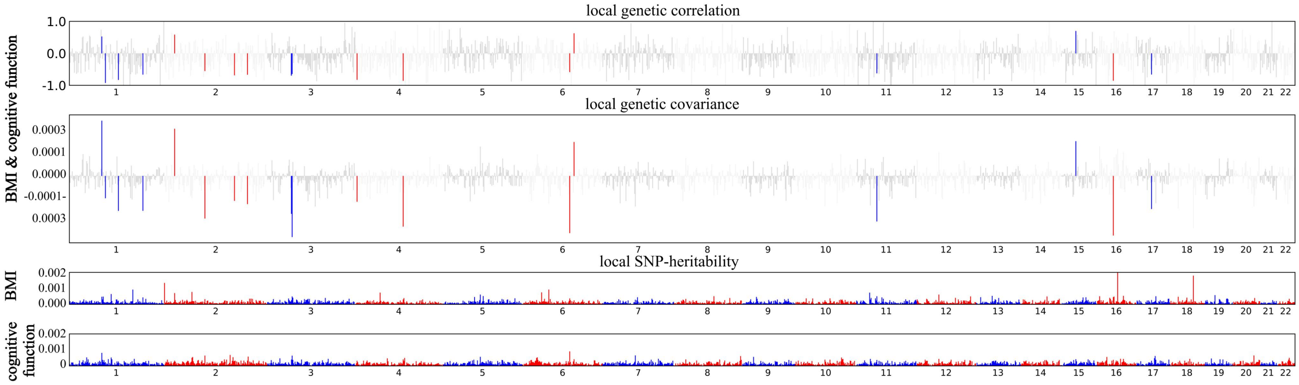

Supplementary Figure 1. Local genetic correlation and covariance between cognitive function and BMI.

The Manhattan plot presents the estimated local genetic correlation and local genetic covariance between cognitive function and BMI, as well as the local SNP heritability for both traits. The red and blue bars within the 'local genetic correlation' and 'local genetic covariance' sections denote significant regions that exhibit shared SNP heritability, having been adjusted for multiple comparisons ($P < 5E{-}08$ for both the local SNP heritability test and $P < 0.05/1700$ for the local genetic covariance test). Trait 1 corresponds to BMI, and Trait 2 refers to cognitive function. BMI: body mass index.

A

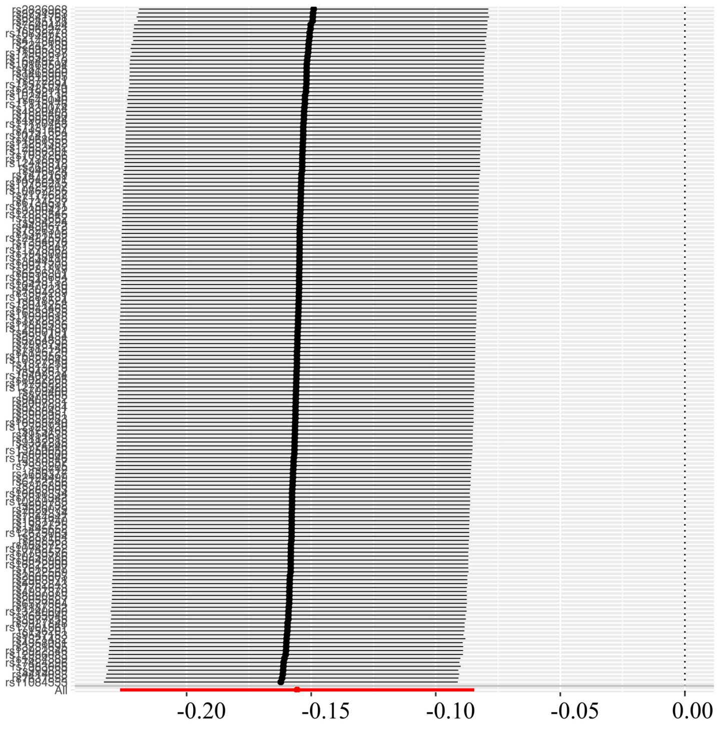

MR leave-one-out sensitivity analysis for ‘BMI’on ‘cognitive function’

B

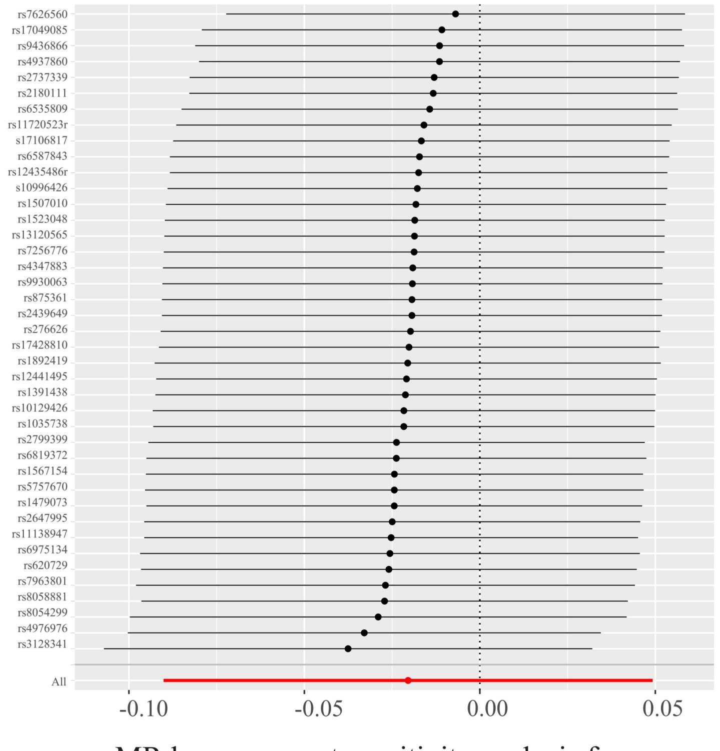

MR leave-one-out sensitivity analysis for ‘cognitive function’ on ‘BMI’

Supplementary Figure 2. Forest plot of leave-one-out analysis for investigating the associations between BMI and cognitive function.

(**A**) The impact of BMI on cognitive function; (**B**) The impact of cognitive function on BMI. Each point in the forest plot represents the association estimate from a single model, with the leave-one-out approach removing one data point at a time to assess the stability of the association. The size of the points corresponds to the weight of the respective data points in the analysis. BMI: body mass index.

A

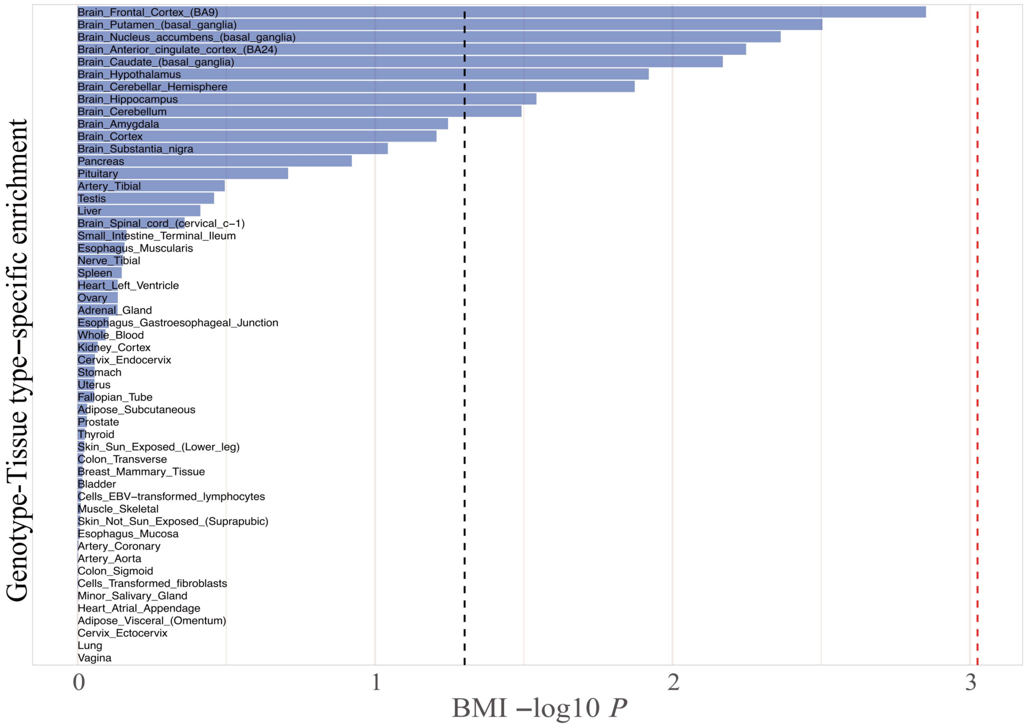

Brain_Frontal_Cortex_(BA9)
Brain_Putamen_(basal_ganglia)
Brain_Nucleus_accumbens_(basal_ganglia)
Brain_Anterior_cingulate_cortex_(BA24)
Brain_Caudate_(basal_ganglia)
Brain_Hypothalamus
Brain_Cerebellar_Hemisphere
Brain_Hippocampus
Brain_Cerebellum
Brain_Amygdala
Brain_Cortex
Brain_Substantia_nigra
Pancreas
Pituitary
Artery_Tibial
Testis
Liver
Brain_Spinal_cord_(cervical_c−1)
Small_Intestine_Terminal_Ileum
Esophagus_Muscularis
Nerve_Tibial
Spleen
Heart_Left_Ventricle
Ovary
Adrenal_Gland
Esophagus_Gastroesophageal_Junction
Whole_Blood
Kidney_Cortex
Cervix_Endocervix
Stomach
Uterus
Fallopian_Tube
Adipose_Subcutaneous
Prostate
Thyroid
Skin_Sun_Exposed_(Lower_leg)
Colon_Transverse
Breast_Mammary_Tissue
Bladder
Cells_EBV−transformed_lymphocytes
Muscle_Skeletal
Skin_Not_Sun_Exposed_(Suprapubic)
Esophagus_Mucosa
Artery_Coronary
Artery_Aorta
Colon_Sigmoid
Cells_Transformed_fibroblasts
Minor_Salivary_Gland
Heart_Atrial_Appendage
Adipose_Visceral_(Omentum)
Cervix_Ectocervix
Lung
Vagina
Genotype-Tissue type−specific enrichment
0
1
2
3
BMI −log10 P

B

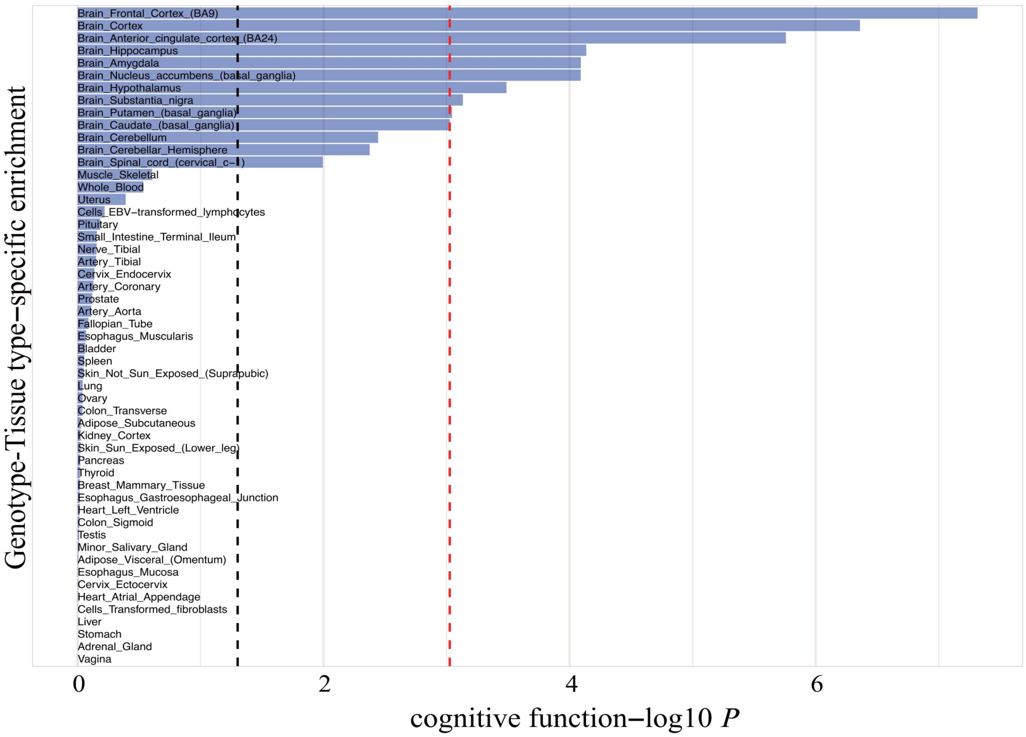

Brain_Frontal_Cortex_(BA9)
Brain_Cortex
Brain_Anterior_cingulate_cortex_(BA24)
Brain_Hippocampus
Brain_Amygdala
Brain_Nucleus_accumbens_(basal_ganglia)
Brain_Hypothalamus
Brain_Substantia_nigra
Brain_Putamen_(basal_ganglia)
Brain_Caudate_(basal_ganglia)
Brain_Cerebellum
Brain_Cerebellar_Hemisphere
Brain_Spinal_cord_(cervical_c−1)
Muscle_Skeletal
Whole_Blood
Uterus
Cells_EBV−transformed_lymphocytes
Pituitary
Small_Intestine_Terminal_Ileum
Nerve_Tibial
Artery_Tibial
Cervix_Endocervix
Artery_Coronary
Prostate
Artery_Aorta
Fallopian_Tube
Esophagus_Muscularis
Bladder
Spleen
Skin_Not_Sun_Exposed_(Suprapubic)
Lung
Ovary
Colon_Transverse
Adipose_Subcutaneous
Kidney_Cortex
Skin_Sun_Exposed_(Lower_leg)
Pancreas
Thyroid
Breast_Mammary_Tissue
Esophagus_Gastroesophageal_Junction
Heart_Left_Ventricle
Colon_Sigmoid
Testis
Minor_Salivary_Gland
Adipose_Visceral_(Omentum)
Esophagus_Mucosa
Cervix_Ectocervix
Heart_Atrial_Appendage
Cells_Transformed_fibroblasts
Liver
Stomach
Adrenal_Gland
Vagina
Genotype-Tissue type−specific enrichment
0
2
4
6
cognitive function−log10 P

Supplementary Figure 3. Stratified linkage disequilibrium score regression (S-LDSC)-based heritability enrichment analysis.

Heritability enrichment analysis across 53 GT3x tissues are presented for (**A**) BMI and (**B**) cognitive function. The X-axis displays the negative log10 p-values of the coefficient Z-scores derived from each individual two-tailed Z-test. The black and red dotted lines indicate the thresholds for statistical significance at FDR of <5% and after Bonferroni correction for multiple comparisons, respectively. BMI: body mass index; GTEx: Genotype-Tissue Expression; FDR: false discovery rate.

A

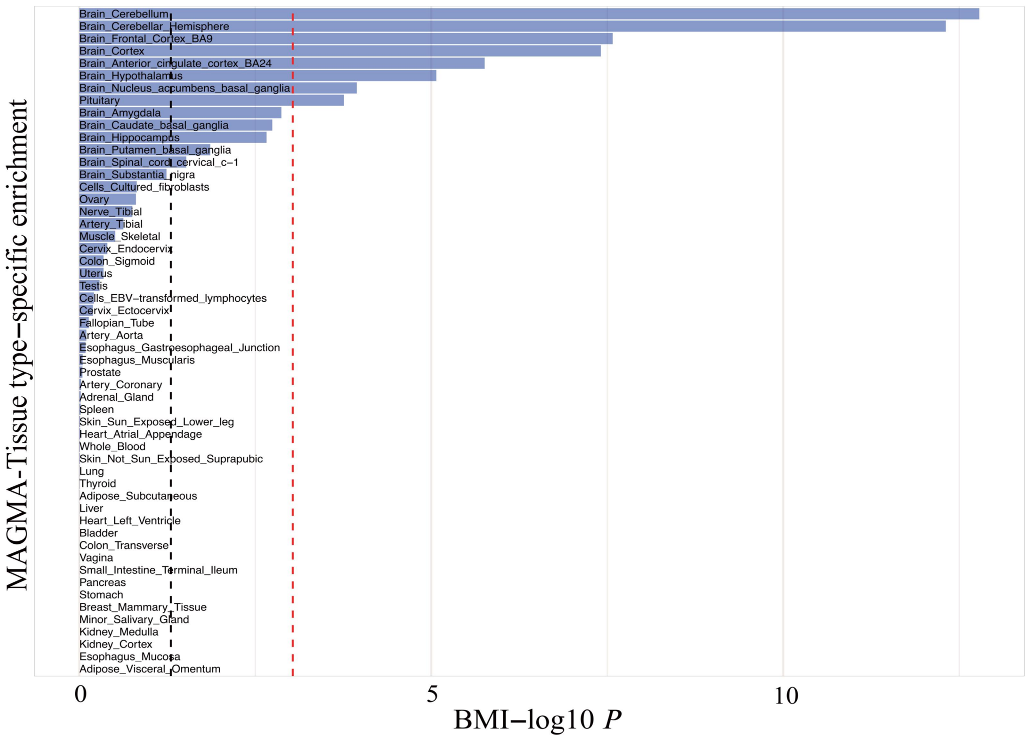
MAGMA-Tissue type−specific enrichment
Brain_Cerebellum
Brain_Cerebellar_Hemisphere
Brain_Frontal_Cortex_BA9
Brain_Cortex
Brain_Anterior_cingulate_cortex_BA24
Brain_Hypothalamus
Brain_Nucleus_accumbens_basal_ganglia
Pituitary
Brain_Amygdala
Brain_Caudate_basal_ganglia
Brain_Hippocampus
Brain_Putamen_basal_ganglia
Brain_Spinal_cord_cervical_c−1
Brain_Substantia_nigra
Cells_Cultured_fibroblasts
Ovary
Nerve_Tibial
Artery_Tibial
Muscle_Skeletal
Cervix_Endocervix
Colon_Sigmoid
Uterus
Testis
Cells_EBV−transformed_lymphocytes
Cervix_Ectocervix
Fallopian_Tube
Artery_Aorta
Esophagus_Gastroesophageal_Junction
Esophagus_Muscularis
Prostate
Artery_Coronary
Adrenal_Gland
Spleen
Skin_Sun_Exposed_Lower_leg
Heart_Atrial_Appendage
Whole_Blood
Skin_Not_Sun_Exposed_Suprapubic
Lung
Thyroid
Adipose_Subcutaneous
Liver
Heart_Left_Ventricle
Bladder
Colon_Transverse
Vagina
Small_Intestine_Terminal_Ileum
Pancreas
Stomach
Breast_Mammary_Tissue
Minor_Salivary_Gland
Kidney_Medulla
Kidney_Cortex
Esophagus_Mucosa
Adipose_Visceral_Omentum
0
5
10
BMI−log10 P

B

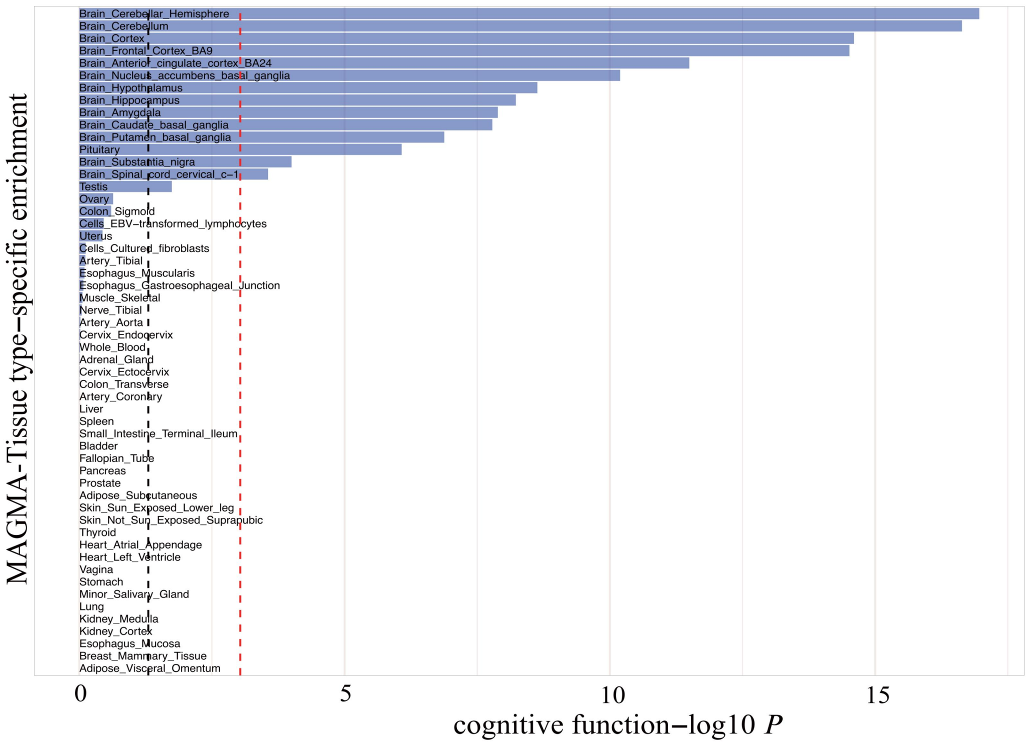
MAGMA-Tissue type−specific enrichment
Brain_Cerebellar_Hemisphere
Brain_Cerebellum
Brain_Cortex
Brain_Frontal_Cortex_BA9
Brain_Anterior_cingulate_cortex_BA24
Brain_Nucleus_accumbens_basal_ganglia
Brain_Hypothalamus
Brain_Hippocampus
Brain_Amygdala
Brain_Caudate_basal_ganglia
Brain_Putamen_basal_ganglia
Pituitary
Brain_Substantia_nigra
Brain_Spinal_cord_cervical_c−1
Testis
Ovary
Colon_Sigmoid
Cells_EBV−transformed_lymphocytes
Uterus
Cells_Cultured_fibroblasts
Artery_Tibial
Esophagus_Muscularis
Esophagus_Gastroesophageal_Junction
Muscle_Skeletal
Nerve_Tibial
Artery_Aorta
Cervix_Endocervix
Whole_Blood
Adrenal_Gland
Cervix_Ectocervix
Colon_Transverse
Artery_Coronary
Liver
Spleen
Small_Intestine_Terminal_Ileum
Bladder
Fallopian_Tube
Pancreas
Prostate
Adipose_Subcutaneous
Skin_Sun_Exposed_Lower_leg
Skin_Not_Sun_Exposed_Suprapubic
Thyroid
Heart_Atrial_Appendage
Heart_Left_Ventricle
Vagina
Stomach
Minor_Salivary_Gland
Lung
Kidney_Medulla
Kidney_Cortex
Esophagus_Mucosa
Breast_Mammary_Tissue
Adipose_Visceral_Omentum
0
5
10
15
cognitive function−log10 P

Supplementary Figure 4. Multi-marker analysis of GenoMic Annotation (MAGMA) -based heritability enrichment analysis.

Heritability enrichment estimates using MAGMA are shown for (**A**) BMI and (**B**) cognitive function. The X-axis plots the negative log10 p-values of the coefficient Z-scores from each individual two-tailed Z-test. The black and red dotted lines signify the thresholds for significance at FDR <5% and following Bonferroni correction for multiple testing, respectively. BMI: body mass index; FDR: false discovery rate.

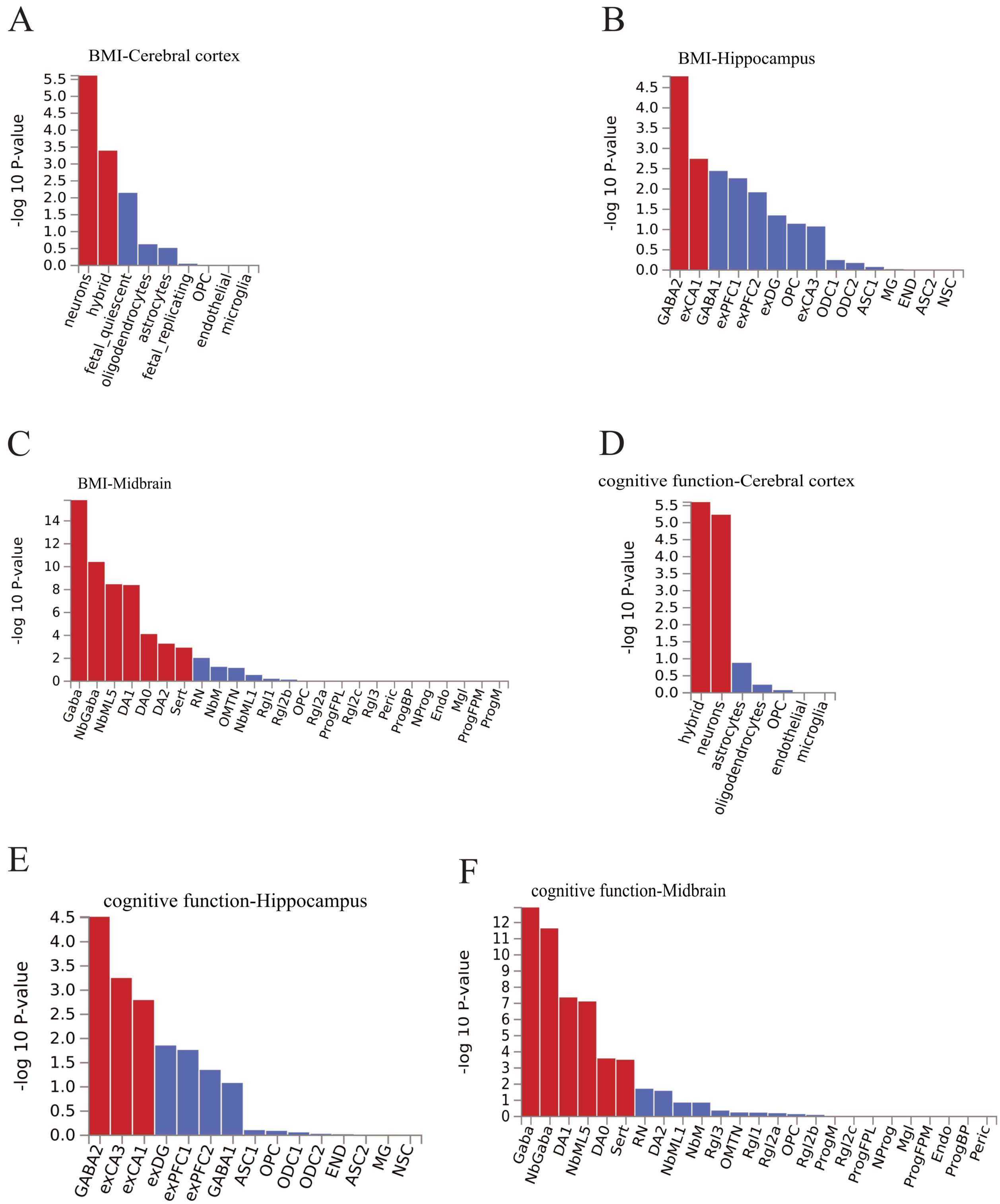

Supplementary Figure 5. Cell-specific SNP heritability enrichment.

The differential enrichment patterns of genetic capacities pertaining to BMI and cognitive function SNPs was shown across various cell types. The cell-specific enrichment associated with BMI within distinct anatomical

regions: (**A**) cerebral cortex, (**B**) hippocampus, and (**C**) midbrain was presented. The cell enrichment linked to cognitive function within the same tissues: (**D**) cerebral cortex, (**E**) hippocampus, and (**F**) midbrain was also identified. The enrichment status is indicated by the color red, which signifies an increase in genetic capacity, whereas blue denotes depletion, indicating a reduction in genetic capacity.
