# Supplementary material for: Investigating causality and shared genetic architecture between body mass index and cognitive function: a genome-wide cross-trait analysis and bi-directional Mendelian randomization study
Source: Front Aging Neurosci. 2024 Oct 16;16:1466799. doi: 10.3389/fnagi.2024.1466799 (PMC11522962; doi:10.3389/fnagi.2024.1466799)
Supplement: Supplementary file 3 [file Data_Sheet_1.docx]

**STROBE-MR checklist of recommended items to address in reports of Mendelian randomization studies**^1^ ^2^

| **Item No.** | **Section** | **Checklist item** | **Page No.** | **Relevant text from manuscript** |
| --- | --- | --- | --- | --- |
| 1 | **TITLE and ABSTRACT** | Indicate Mendelian randomization (MR) as the study’s design in the title and/or the abstract if that is a main purpose of the study | Article | Investigating causality and shared genetic architecture between body mass index and cognitive function: a genome-wide cross-trait analysis and bi-directional Mendelian randomization study |
|  | **INTRODUCTION** |  |  |  |
| 2 | **Background** | Explain the scientific background and rationale for the reported study. What is the exposure? Is a potential causal relationship between exposure and outcome plausible? Justify why MR is a helpful method to address the study question | Article | Observational studies have established a connection between body mass index (BMI) and an increased risk of cognitive decline. However, a comprehensive investigation into the causal relationships between BMI and cognitive function across diverse age groups, as well as the genetic underpinnings of this relationship, has been notably lacking. |
| 3 | **Objectives** | State specific objectives clearly, including pre-specified causal hypotheses (if any). State that MR is a method that, under specific assumptions, intends to estimate causal effects | Article | This study aims to investigate causality and the shared genetic underpinnings of between BMI and cognitive function by conducting a thorough genome-wide analysis, thereby provide valuable insights for developing personalized intervention strategies to promote cognitive health. |
|  | **METHODS** |  |  |  |
| 4 | **Study design and data sources** | Present key elements of the study design early in the article. Consider including a table listing sources of data for all phases of the study. For each data source contributing to the analysis, describe the following: |  |  |
|  | a) | Setting: Describe the study design and the underlying population, if possible. Describe the setting, locations, and relevant dates, including periods of recruitment, exposure, follow-up, and data collection, when available. | Article | 2.1 Study design, data summary and quality control |
|  | b) | Participants: Give the eligibility criteria, and the sources and methods of selection of participants. Report the sample size, and whether any power or sample size calculations were carried out prior to the main analysis | Supplementary | Supplementary Table 1  Supplementary Table 2 |
|  | c) | Describe measurement, quality control and selection of genetic variants | Article | 2.2 Heritability and genetic correlation |
|  | d) | For each exposure, outcome, and other relevant variables, describe methods of assessment and diagnostic criteria for diseases | Article | 2.2 Heritability and genetic correlation |
|  | e) | Provide details of ethics committee approval and participant informed consent, if relevant | / | / |
| 5 | **Assumptions** | Explicitly state the three core IV assumptions for the main analysis (relevance, independence and exclusion restriction) as well assumptions for any additional or sensitivity analysis | Article | 2.7 Mendelian randomization analysis |
| 6 | **Statistical methods: main analysis** | Describe statistical methods and statistics used |  |  |
|  | a) | Describe how quantitative variables were handled in the analyses (i.e., scale, units, model) | Article | 2.2 Heritability and genetic correlation |
|  | b) | Describe how genetic variants were handled in the analyses and, if applicable, how their weights were selected | Article | 2.2 Heritability and genetic correlation |
|  | c) | Describe the MR estimator (e.g. two-stage least squares, Wald ratio) and related statistics. Detail the included covariates and, in case of two-sample MR, whether the same covariate set was used for adjustment in the two samples | Article | 2.7 Mendelian randomization analysis |
|  | d) | Explain how missing data were addressed | / | / |
|  | e) | If applicable, indicate how multiple testing was addressed | / | / |
| 7 | **Assessment of assumptions** | Describe any methods or prior knowledge used to assess the assumptions or justify their validity | Article | 2.7 Mendelian randomization analysis |
| 8 | **Sensitivity analyses and additional analyses** | Describe any sensitivity analyses or additional analyses performed (e.g. comparison of effect estimates from different approaches, independent replication, bias analytic techniques, validation of instruments, simulations) | Article | 2.7 Mendelian randomization analysis |
| 9 | **Software and pre-registration** |  |  |  |
|  | a) | Name statistical software and package(s), including version and settings used | Article | 2.7 Mendelian randomization analysis |
|  | b) | State whether the study protocol and details were pre-registered (as well as when and where) | Article | 2.7 Mendelian randomization analysis |
|  | **RESULTS** |  |  |  |
| 10 | **Descriptive data** |  |  |  |
|  | a) | Report the numbers of individuals at each stage of included studies and reasons for exclusion. Consider use of a flow diagram | Article | Figure 1 |
|  | b) | Report summary statistics for phenotypic exposure(s), outcome(s), and other relevant variables (e.g. means, SDs, proportions) | Supplementary | Supplementary Table 1  Supplementary Table 2 |
|  | c) | If the data sources include meta-analyses of previous studies, provide the assessments of heterogeneity across these studies | / | / |
|  | d) | For two-sample MR:  i.  Provide justification of the similarity of the genetic variant-exposure associations between the exposure and outcome samples  ii.  Provide information on the number of individuals who overlap between the exposure and outcome studies | Article | 2.2 Heritability and genetic correlation |
| 11 | **Main results** |  |  |  |
|  | a) | Report the associations between genetic variant and exposure, and between genetic variant and outcome, preferably on an interpretable scale | Supplementary | Supplementary Table 8 |
|  | b) | Report MR estimates of the relationship between exposure and outcome, and the measures of uncertainty from the MR analysis, on an interpretable scale, such as odds ratio or relative risk per SD difference | Supplementary | Supplementary Figure 2 |
|  | c) | If relevant, consider translating estimates of relative risk into absolute risk for a meaningful time period | / | / |
|  | d) | Consider plots to visualize results (e.g. forest plot, scatterplot of associations between genetic variants and outcome versus between genetic variants and exposure) | Article | Figure 2 |
| 12 | **Assessment of assumptions** |  |  |  |
|  | a) | Report the assessment of the validity of the assumptions | / | / |
|  | b) | Report any additional statistics (e.g., assessments of heterogeneity across genetic variants, such as *I^2^*, Q statistic or E-value) | Supplementary | Supplementary Table 8 |
| 13 | **Sensitivity analyses and additional analyses** |  |  |  |
|  | a) | Report any sensitivity analyses to assess the robustness of the main results to violations of the assumptions | / | Figure 2 |
|  | b) | Report results from other sensitivity analyses or additional analyses | / | Figure 2 |
|  | c) | Report any assessment of direction of causal relationship (e.g., bidirectional MR) | / | Figure 2 |
|  | d) | When relevant, report and compare with estimates from non-MR analyses | / | / |
|  | e) | Consider additional plots to visualize results (e.g., leave-one-out analyses) | Supplementary | Supplementary Table 8  Supplementary Figure 2 |
|  | **DISCUSSION** |  |  |  |
| 14 | **Key results** | Summarize key results with reference to study objectives | Article | A significant negative correlation was found between BMI and cognitive function (β = -0.16, P = 1.76E-05). Cross-trait meta-analysis and SMR identified two novel risk genes, TUFM and MST1R, which had not been found in previous studies. Significant enrichment of single nucleotide polymorphisms (SNP) for BMI and cognitive function was found at tissue and cell-specific levels, mainly within the brain. |
| 15 | **Limitations** | Discuss limitations of the study, taking into account the validity of the IV assumptions, other sources of potential bias, and imprecision. Discuss both direction and magnitude of any potential bias and any efforts to address them | Article | We acknowledge certain limitations in our research. Initially, our findings are confined to European populations, necessitating future studies to expand the scope to include other ancestral groups to comprehensively uncover the biological mechanisms at play in these two traits. Furthermore, our investigation did not delve into the etiology of cognitive function, suggesting a need for larger GWAS datasets focusing on BMI and cognitive function to validate our results across diverse ethnicities. Additionally, our assessment of tissue enrichment was based on the 54 tissue types available in the GTEx database, which may restrict our understanding of gene regulatory mechanisms due to the limited range of tissues examined. |
| 16 | **Interpretation** |  |  |  |
|  | a) | Meaning: Give a cautious overall interpretation of results in the context of their limitations and in comparison with other studies | Article | In conclusion, this study has unveiled a negative correlation between BMI and cognitive function across different age populations, identifying TUFM and MST1R as novel genetic markers that influence both BMI and cognitive function. This critical discovery not only enriches our understanding of the physiological underpinnings of both obesity and cognitive decline, but also offers crucial insights for the development of preventive strategies to counteract cognitive deterioration in individuals with obesity. |
|  | b) | Mechanism: Discuss underlying biological mechanisms that could drive a potential causal relationship between the investigated exposure and the outcome, and whether the gene-environment equivalence assumption is reasonable. Use causal language carefully, clarifying that IV estimates may provide causal effects only under certain assumptions | Article | While numerous studies have explored the connection between Body Mass Index (BMI) and cognitive function, the BMI’s effect on cognitive function remains a subject of debate. Some research has indicated a correlation between a higher BMI and a reduced risk of cognitive decline in the late-life and elderly populations, suggesting that older individuals who are overweight might be less prone to cognitive impairment(Cronk, Johnson, and Burns 2010; Sobów, Fendler, and Magierski 2014). For instance, a study that tracked individuals over time found that a BMI of over 25.0 was associated with a lower risk of early dementia, with weight loss being identified as an early indicator of the condition(Atti et al. 2008). Additionally, the study noted that unexplained weight loss in older individuals was associated with diminished cognitive function. Furthermore, a longitudinal cohort study with an 8-year follow-up duration reported a negative correlation between BMI and cognitive decline among elderly participants. The study found that each one-unit increase in BMI score was associated with an 8% reduction in the risk of dementia, which suggests that older adults with higher BMI scores are at lesser risk of developing dementia(Dahl et al. 2008). In the other hand, higher BMI was found to be associated with cognitive decline in mid-life and younger populations(Meo et al. 2019; West et al. 2021). Therefore, the relationship between BMI and cognitive function at the population level across different age groups remains elusive. Furthermore, the extent to which genetic factors contribute to a shared risk profile for BMI and cognitive function has not been adequately examined in previous studies. In response to this knowledge gap, we aimed to conduct an extensive investigation using genome-wide association studies (GWAS) summary-level data to uncover potential genetic associations that might connect BMI and cognitive function. |
|  | c) | Clinical relevance: Discuss whether the results have clinical or public policy relevance, and to what extent they inform effect sizes of possible interventions | Article | This discovery not only enriches our understanding of the physiological underpinnings of both obesity and cognitive decline, but also offers crucial insights for the development of preventive strategies to counteract cognitive deterioration in individuals with obesity. |
| 17 | **Generalizability** | Discuss the generalizability of the study results (a) to other populations, (b) across other exposure periods/timings, and (c) across other levels of exposure | Article | In conclusion, this study has unveiled a negative correlation between BMI and cognitive function across different age populations, identifying TUFM and MST1R as novel genetic markers that influence both BMI and cognitive function. This critical discovery not only enriches our understanding of the physiological underpinnings of both obesity and cognitive decline, but also offers crucial insights for the development of preventive strategies to counteract cognitive deterioration in individuals with obesity. |
|  | **OTHER INFORMATION** |  |  |  |
| 18 | **Funding** | Describe sources of funding and the role of funders in the present study and, if applicable, sources of funding for the databases and original study or studies on which the present study is based | Article | This work was funded by grants from Funding by Science and Technology Projects in Guangzhou (grant number 2024A03J0818) and Guangzhou Science and Technology Plan Project (grant number 2024A03J0818). |
| 19 | **Data and data sharing** | Provide the data used to perform all analyses or report where and how the data can be accessed, and reference these sources in the article. Provide the statistical code needed to reproduce the results in the article, or report whether the code is publicly accessible and if so, where | Article | 2.1 Study design, data summary and quality control |
| 20 | **Conflicts of Interest** | All authors should declare all potential conflicts of interest | Article | The authors declare that the research was conducted in the absence of any commercial or financial relationships that could be construed as a potential conflict of interest. |

This checklist is copyrighted by the Equator Network under the Creative Commons Attribution 3.0 Unported (CC BY 3.0) license.

1. Skrivankova VW, Richmond RC, Woolf BAR, Yarmolinsky J, Davies NM, Swanson SA, et al. Strengthening the Reporting of Observational Studies in Epidemiology using Mendelian Randomization (STROBE-MR) Statement. JAMA. 2021;under review.

2. Skrivankova VW, Richmond RC, Woolf BAR, Davies NM, Swanson SA, VanderWeele TJ, et al. Strengthening the Reporting of Observational Studies in Epidemiology using Mendelian Randomisation (STROBE-MR): Explanation and Elaboration. BMJ. 2021;375:n2233.
